# Dissecting Stages of Human Kidney Development and Tumorigenesis with Surface Markers Affords Simple Prospective Purification of Nephron Stem Cells

**DOI:** 10.1038/srep23562

**Published:** 2016-03-29

**Authors:** Naomi Pode-Shakked, Oren Pleniceanu, Rotem Gershon, Rachel Shukrun, Itamar Kanter, Efrat Bucris, Ben Pode-Shakked, Gal Tam, Hadar Tam, Revital Caspi, Sara Pri-Chen, Einav Vax, Guy Katz, Dorit Omer, Orit Harari-Steinberg, Tomer Kalisky, Benjamin Dekel

**Affiliations:** 1 Pediatric Stem Cell Research Institute, Edmond and Lily Safra Children’s Hospital, Sheba Medical Center, Tel-Hashomer, Israel; 2 Sheba Centers for Regenerative Medicine and Cancer Research, Sheba Medical Center, Tel-Hashomer, Israel; 3 The Dr. Pinchas Borenstein Talpiot Medical Leadership Program, Sheba Medical Center, Tel-Hashomer, Israel; 4 Faculty of Engineering and Bar-Ilan Institute of Nanotechnology and Advanced Materials (BINA), Bar-Ilan University, Ramat Gan, Israel; 5 The Danek Gertner Institute of Human Genetics, Sheba Medical Center, Tel-Hashomer, Israel; 6 The Maurice and Gabriela Goldschleger Eye Research Institute, Sheba Medical Center, Tel-Hashomer, Israel; 7 The Joseph Buchman Gynecology and Maternity Center, Sheba Medical Center, Tel-Hashomer, Israel; 8 Division of Pediatric Nephrology, Edmond and Lily Safra Children’s Hospital, Sheba Medical Center, Tel-Hashomer, Israel; 9 Sackler Faculty of Medicine, Tel-Aviv University, Tel-Aviv, Israel

## Abstract

When assembling a nephron during development a multipotent stem cell pool becomes restricted as differentiation ensues. A faulty differentiation arrest in this process leads to transformation and initiation of a Wilms’ tumor. Mapping these transitions with respective surface markers affords accessibility to specific cell subpopulations. NCAM1 and CD133 have been previously suggested to mark human renal progenitor populations. Herein, using cell sorting, RNA sequencing, *in vitro* studies with serum-free media and *in vivo* xenotransplantation we demonstrate a sequential map that links human kidney development and tumorigenesis; In nephrogenesis, NCAM1^+^CD133^−^ marks SIX2^+^ multipotent renal stem cells transiting to NCAM1^+^CD133^+^ differentiating segment-specific SIX2^−^ epithelial progenitors and NCAM1^−^CD133^+^ differentiated nephron cells. In tumorigenesis, NCAM1^+^CD133^−^ marks SIX2^+^ blastema that includes the ALDH1^+^ WT cancer stem/initiating cells, while NCAM1^+^CD133^+^ and NCAM1^−^CD133^+^ specifying early and late epithelial differentiation, are severely restricted in tumor initiation capacity and tumor self-renewal. Thus, negative selection for CD133 is required for defining NCAM1^+^ nephron stem cells in normal and malignant nephrogenesis.

The mammalian kidney is formed via reciprocally inductive interactions between two mesodermal precursor tissues, namely the metanephric mesenchyme (MM) and ureteric bud^1^. A subpopulation of MM cells, most adjacent to UB tips, termed cap mesenchyme (CM) cells, represents the pool of multipotent renal stem cells, as they both self-renew and give rise to different types of nephron epithelia^2^ via a process of mesenchymal to epithelial transition (MET). The CM population expresses a unique combination of transcription factors, including SIX2 and WT1, which are considered early markers of kidney progenitors. The ultimate goal of renal regenerative medicine is to isolate and/or create an unlimited supply of human cells resembling the renal progenitors residing in the CM, harboring true nephrogenic potential, in order to regenerate and replenish epithelial cell types within the nephron. Recently, we demonstrated that isolation of NCAM1^+^ hFK cells grown in serum-free culture selects for SIX2-epressing CM cells, representing a mitotically active cell population harboring stem/progenitor traits, including enhanced clonogenic and renal differentiation capacity and therapeutic potential in a 5/6 nephrectomy kidney injury model. However, NCAM1^+^ cells selected according to this methodology were composed of both stem SIX2^+^ and progenitor SIX2^−^ cells, which could not be separated. Thus, while the transcriptional profile of the CM has been thoroughly characterized, a concomitant surface marker expression pattern, which could allow prospective isolation of this population is still lacking. Interestingly, the pediatric renal malignancy Wilms’ tumor (WT), which is the most common pediatric tumor of the kidney, accounting for approximately 7% of childhood cancers^3^, is thought to originate from the aberrant version of the same CM cells^4^,^5^,^6^. Accordingly, WT is typically composed of three compartments reminiscent of the normal hFK: Undifferentiated blastema, interstitial compartment and epithelial compartment, corresponding to hFK CM, interstitium and tubular epithelia, respectively^1^,^7^. Hence, both the normal and aberrant renal differentiation seen during embryonic nephrogenesis and WT carcinogenesis, respectively, can be envisioned as taking place along an MET (mesenchymal to epithelial transition) differentiation axis. Situated at the top of this axis in the hFK is the CM population, representing the pre-MET stage. Accordingly, the post-MET structures are defined as epithelial structures emerging after the commencement of epithelial differentiation^8^. These include both early post-MET structures (e.g. pre-tubular aggregates and C/S-shaped bodies), which have undergone only partial differentiation, and fully differentiated renal tubules. Notably, unlike the pre-MET stage, during the post-MET stages new renal cells are generated from unipotent precursors, restricted within the boundaries of a specific tubular segment^8^. In parallel, WT development includes transformed renal progenitors that maintain themselves as undifferentiated blastema (pre-MET stage) while at the same time aberrantly differentiating first into tubular elements^5^,^9^ in various degrees of maturation, including both immature tubules (early post-MET) and differentiated epithelia (late post-MET). We previously used global gene expression analysis of various differentiated (adult kidneys and renal cell carcinoma) and undifferentiated (hFK, human WT and patient derived WT xenografts [WT PDX]) renal tissues, to elucidate putative surface markers that could allow isolation of renal progenitor populations^10^,^11^,^12^,^13^. We identified NCAM1 as a marker of both normal and malignant renal stem cells^12^,^13^. A second marker shown to be over-expressed in renal progenitor-rich tissues was FZD7, previously shown to play a role in normal and cancer stem cell function. However, sorting according to FZD7 resulted in extensive cell death, precluding it from serving as a selection marker for malignant renal progenitor cells^12^,^13^. In contrast, selection according to CD133, which has been shown to represent a CSC marker in several malignancies^14^,^15^,^16^,^17^,^18^,^19^, did not enrich for cells with progenitor properties^13^,^20^,^21^. Accordingly, using WT-PDX we recently showed that the WT CSCs are NCAM1^+^ALDH1^+^1^+^ cells, which are exclusively localized within the WT blastema^20^. More recently, we utilized a pure-blastema WT-PDX model and found that the homogenous-appearing SIX2^+^NCAM1^+^ blastema is actually a heterogeneous population that follows a renal differentiation gradient (containing high and lower expression domains of SIX2). Within this gradient, we showed that the ALDH1^+^WT CSCs are not the most primitive cell type in terms of renal differentiation. Instead, within the blastema they are slightly skewed towards epithelial commitment, as manifested by a slight decline in expression of renal progenitor genes (e.g. *SIX2 and WT1)* and relatively higher levels of epithelial markers. Thus, this work established a model of WT propagation, in which WT CSCs, which actively sustain tumor growth, transit between mesenchyme and epithelia to de-differentiate into earlier SIX2-high blastemal cells as well as differentiate into mature epithelia^21^. Here, we were interested in identifying a specific surface marker expression pattern of both nephron stem/progenitors in hFK and cancer stem cells in WT, which could allow prospective isolation of the former as well as further characterization of the latter, towards more efficient eradication of the tumor. To achieve this goal, we investigated the expression of NCAM1, FZD7 and CD133 in the various cellular compartments of human fetal kidney (hFK), primary Wilms’ tumor (pWT) and Wilms, tumor patient–derived xenografts (WT-PDX). We show that NCAM1^+^CD133^−^ cells sorted from hFK harbor a primitive, CM-like phenotype, as manifested by renal stem cell signature set, lack of expression of renal maturation markers and multipotent renal differentiation potential, hence representing nephron stem cells. Similarly, we show that a the NCAM1^+^CD133^−^ fraction of primary human WT defines WT blastema and that within this compartment reside WT-CSCs, verifying our findings in the pure blastema WT-PDX model. These findings allow establishment of a more generalized scheme of the various cellular components of hFK and WT, and afford simple method to isolate human nephron stem cells and define CSCs in primary human WT.

## Results

### NCAM1, CD133 and FZD7 define cell lineages in human fetal kidney (hFK) and primary WT (pWT)

**Figure 1:**
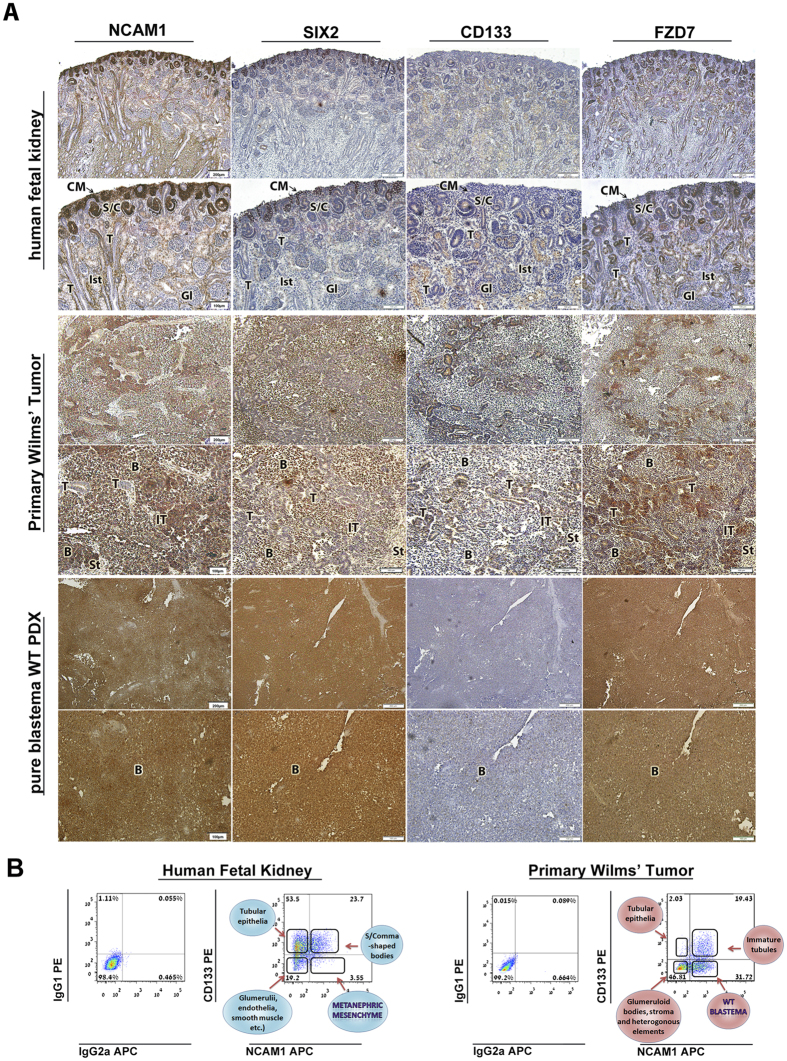
NCAM1, SIX2, CD133 and FZD7 expression defines distinct cellular compartments in human fetal kidney (hFK) and primary WT (pWT) (**A**) Immunohistochemical staining (IHC) for NCAM1, SIX2, CD133 and FZD7 in representative hFK, pWT and pure blastema WT-PDX presented in serial sections. SIX2 is expressed in the cap mesenchyme (CM) and early post-MET structures (e.g. C-/S- shaped bodies) in the hFK and in WT blastema. The NCAM1 expression domain includes the SIX2 expression domain and also the hFK interstitium. In contrast, CD133 is expressed in mature tubular epithelia in both hFK and pWT as well as in early post-MET (S/C) structures in the hFK and immature tubules (IT) in pWT. FZD7 expression spans all cellular compartments except for the hFK interstitium, but in a non-uniform staining pattern. Accordingly, pure blastema WT-PDX uniformly express SIX2, NCAM1 and FZD7 but are devoid of CD133 expression. (**B**) Representative flow cytometry plots of hFK and pWT according to NCAM1 and CD133 expression, delineating the different cellular compartments in these tissues. Thus, cellular lineages along the renal developmental MET axis in hFK and pWT can be defined according to the expression of these markers. T-Tubules; B-Blastema; IT-Immature tubules; St-Stroma; CM-Condensed mesenchyme; S/C-S-shaped/Comma shaped bodies; Ist-Interstitium. (Scale bars are indicated in the images).

In order to identify a specific surface marker expression pattern that could define the different MET-associated cellular compartments in hFK and WT, we initially carried out immunohistochemical staining (IHC) of hFK, primary WT (pWT) and pure blastema WT-patient-derived xenografts (WT PDX) for the surface markers NCAM1, FZD7 and CD133 and the transcription factor SIX2 (a marker of early embryonic renal progenitors) (Fig. 1A, Table 1 and Figure S1). As expected, SIX2 was localized to the progenitor compartments in both hFK (i.e. CM and its early derivatives) and pWT (i.e. undifferentiated blastema). Accordingly, pure blastema WT-PDX were uniformly SIX2^+^. Interestingly, NCAM1 and CD133 demonstrated a reciprocal expression pattern in both hFK and pWT. While NCAM1 localized mainly to the CM, blastema, early post-MET structures (C/S- shaped bodies and immature tubules in hFK and pWT, respectively) and interstitium (only in hFK), CD133 was detected in mature epithelial structures and to a lesser extent in early post-MET structures, but was completely excluded from the CM and blastema. Supporting this notion, pure blastema WT-PDX were entirely NCAM1^+^ but completely devoid of CD133 expression. Finally, FZD7 expression was detected in all cellular compartments of both tissues, except for the hFK interstitium and WT stroma. However, FZD7 staining was not uniform within these compartments, but rather showed a scattered expression pattern within each compartment. We next performed flow cytometry analysis of dissociated hFK and pWT for combinations of NCAM1, CD133 and FZD7 expression. According to their histological localizations, both hFK and pWT could be separated into four distinct cell populations according to the expression of these three surface markers (Fig. 1B, Table 2 and Figure S2): i. NCAM1^+^CD133^−^, corresponding to the undifferentiated CM and blastema and to the renal interstitium in hFK. Importantly, the latter could be excluded via further selection of FZD7^+^ cells. ii. NCAM1^+^CD133^+^, corresponding to early post-MET structures (e.g. C/S- shaped bodies and immature tubules in hFK and pWT, respectively); iii. NCAM1^−^CD133^+^, corresponding to differentiated tubular epithelia; iv. NCAM1^−^CD133^−^, representing various non-renal epithelial lineage kidney compartments. The latter include endothelium, mesangial cells and smooth muscle in hFK and glomeruloid bodies, vessels, stroma and various mesodermal heterologous elements in WT. Taken together, these results indicate that NCAM1 and CD133 display opposite expression patterns along the renal MET axis, in both hFK and WT. While NCAM1 expression is prominent in the undifferentiated, mesenchymal structures and is gradually lost along epithelial differentiation, CD133 expression increases concomitant with renal epithelialization. Importantly, overlapping expression of NCAM1 and CD133 is noted in the early post-MET structures. In contrast, FZD7 is absent only from the interstitium, thereby serving as an exclusion marker for this compartment in hFK. Hence, sorting according to an NCAM1^+^CD133^−^ phenotype in pWT and according to NCAM1^+^CD133^−^FZD7^+^ in hFK, would potentially allow for the isolation of a purified progenitor population from both tissues.

However, our experience had shown that FZD7 is an unreliable marker for sorting of cell subpopulations from hFK and WT, as it both undergoes frequent ligand-receptor complex internalization, and as anti-FZD7 Abs brought induced cell death of significant cell portions^22^. In order to circumvent this problem, we have used a serum free medium (SFM) for culturing hFK cells. Immunofluorescence staining of cells from this cultured population showed them to contain either hFK epithelia (Cytokeratin^+^) or CM (SIX2^+^) but not interstitial cells (Figure S3). This allowed us to specifically isolate the CM using NCAM1 and CD133 alone, without depending on FZD7 for sorting purposes. This notion reinforces our previous observation that SFM culture conditions result in enrichment for the epithelial lineage, while the NCAM1^+^ subpopulation in these conditions appears to be less differentiated, conserving SIX2 expression^23^.

### NCAM1 and CD133 define the mesenchymal to epithelial (MET) hierarchy in human fetal kidney cells

**Figure 2:**
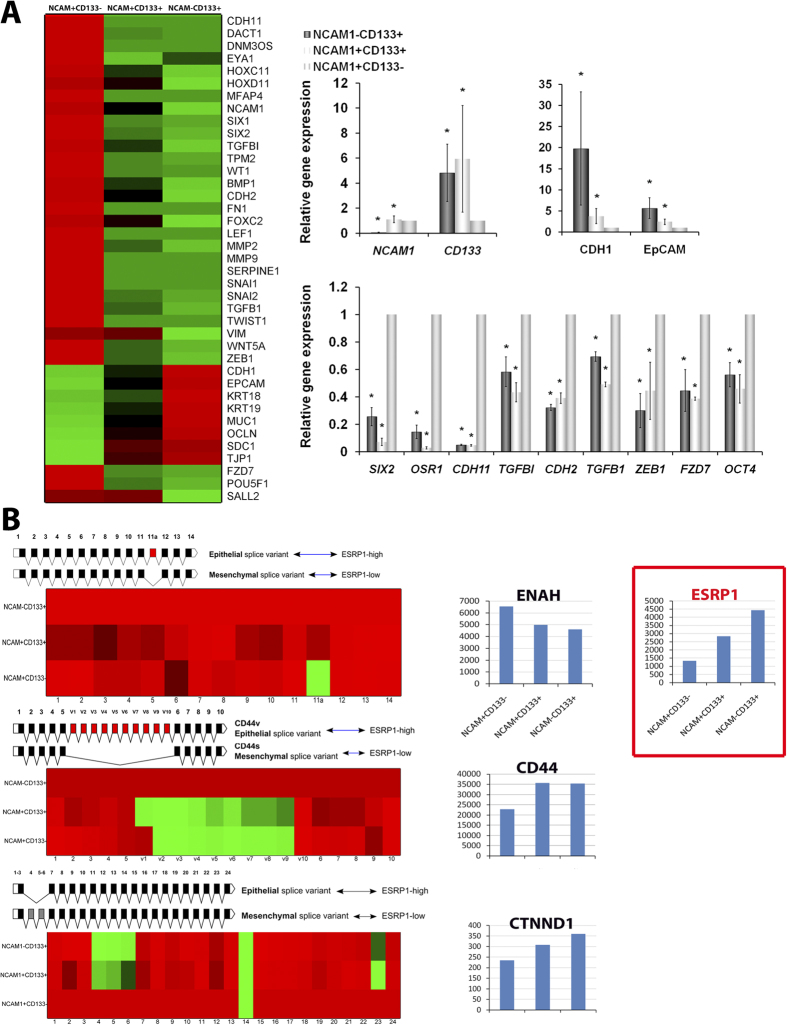
NCAM1 and CD133 define the MET hierarchy in hFK cells. RNA sequencing of hFK cells grown in SFM and sorted into three cell fractions: NCAM1^+^CD133^−^, NCAM1^+^CD133^+^ and NCAM1^−^CD133^+^. (**A**) Left: Heatmap representation of differentially expressed genes between the three cell subpopulations showing cap mesenchyme (CM) and mesenchymal genes to be highly expressed by NCAM1^+^CD133^−^ cells and gradually decline in NCAM1^+^CD133^+^ and NCAM1^−^CD133^+^ hFK cells. In contrast, epithelial genes are most highly expressed by NCAM1^−^CD133^+^, to a lesser extent in NCAM1^+^CD133^+^ cells and are drastically downregulated in NCAM1^+^CD133^−^ cells. Right: validation of RNA sequencing results via qRT-PCR performed on sorted cells from different hFKs showing the same hierarchical MET gene expression pattern between these cell fractions. The values for NCAM1^+^CD133^−^ cells were used to normalize (therefore = 1) and all other values were calculated with respect to them. Experiments were performed on 2 hFK sources (n = 2). Results are presented as the mean ± S.E.M of three separate experiments; *p < 0.05; (**B**) In several genes, splice isoforms that are characteristic of epithelial and mesenchymal states during EMT are differentially expressed during fetal kidney differentiation, consistent with NCAM1 and CD133 expression. Left: For each gene, shown is a diagram representing the alternative splice variants and a heatmap representing the exon inclusion frequency (the relative number of RNA sequencing reads that overlap with each exon; red – high, green - low). Left, upper: Similarly, for ENAH (hMENA), the mesenchymal associated isoform (skipping exon 11a) is over-expressed in the NCAM1^+^CD133^−^ cell population, whereas the epithelial associated isoform is over-expressed in NCAM1^−^CD133^+^ cells. Left, middle: For CD44, the mesenchymal associated isoform (CD44s, excluding exons V1-V10) is over-expressed in the NCAM1^+^CD133^−^ fraction, while the epithelial associated isoform (CD44v) is over-expressed in the NCAM1^−^CD133^+^ fraction. Left, bottom: CTNND1 shows the opposite pattern, where the mesenchymal associated isoform (including exons 4–6) is over-expressed in the NCAM1^+^CD133^−^ population, while the epithelial associated isoform is over-expressed in NCAM1^−^CD133^+^ cells. Right: Consistent with the above, ESRP1, an epithelial splicing regulatory protein that regulates the formation of epithelial cell-specific isoforms during EMT, shows a gradual increase from the NCAM1^+^CD133^−^ to the NCAM1^−^CD133^+^ cell population.

So as to assess whether NCAM1 and CD133 could indeed define distinct populations of hFK in terms of the developmental MET axis, we next carried out global gene expression analysis of sorted cell fractions, using RNA-sequencing. We analyzed three sorted hFK populations: NCAM1^+^CD133^−^, NCAM1^+^CD133^+^ and NCAM1^−^CD133^+^. Interestingly, the three populations demonstrated a distinct gene expression pattern with respect to CM-related, mesenchymal, epithelial and stemness genes (Fig. 2A, left panel and Table 3). NCAM1^+^CD133^−^ exhibited strong expression of a wide array of CM-related (e.g. *EYA1*, *OSR1*, *SIX1* and *SIX2*) and mesenchymal (e.g. *TGFB1*, *TWIST1* and *VIM*) genes. These genes were significantly silenced in NCAM1^+^CD133^+^, and even more so in NCAM1^−^CD133^+^. Stemness genes, such as *OCT4* and *SOX2*, demonstrated a similar expression gradient along the three cell types. Conversely, epithelial genes (e.g. *EpCAM*, *MUC1, CDH1* and *SDC1*) demonstrated strong expression in NCAM1^−^CD133^+^ cells, intermediate expression in NCAM1^+^CD133^+^ cells and very low expression in NCAM1^+^CD133^−^ cells. These findings were validated via qPCR analysis of representative genes in the three cell fractions (Fig. 2A, right panel). In line with the RNA-seq results, we found opposite expression gradients of CM-related, mesenchymal and stemness genes (maximal in NCAM1^+^CD133^−^ cells) and epithelial genes (maximal in NCAM1^−^CD133^+^ cells). Furthermore, single cell qPCR gene expression measurements showed NCAM1 and CD133 to mark different cell fractions in the human fetal kidney. The CD133^+^ fraction (but not the NCAM1^+^ fraction) expresses epithelial markers such as CDH1 (E-Cadherin) (Figure S4). Likewise, we also observed splice isoform switching in several genes (CD44, ENAH, and CTNND1, Fig. 2B and Fig. S5) between the three hFK cell populations, which are consistent with previous observations of EMT progression during embryonic development and cancer^24^,^25^,^26^,^27^,^28^,^29^.

We observed gradual progression from mesenchymal- to epithelial-associated isoforms, such that the NCAM1^+^CD133^−^ fraction overexpressed mesenchymal-associated isoforms, NCAM1^+^CD133^−^ cells expressed an intermediate mixture of both isoforms, and NCAM1^−^CD133^+^ cells overexpressed epithelial-associated isoforms (Fig. 2B, left). Furthermore, Epithelial Regulator of Splicing 1 (ESRP1) was found to gradually increase between NCAM1^+^CD133^−^, NCAM1^+^CD133^+^ cells and NCAM1^−^CD133^+^ fractions (Fig. 2B, right), suggesting that ESRP1 may play a major role in mRNA splicing regulation during human nephrogenic MET.

### Immunostaining reveals an undifferentiated phenotype of NCAM1^+^CD133^−^ cells

**Figure 3:**
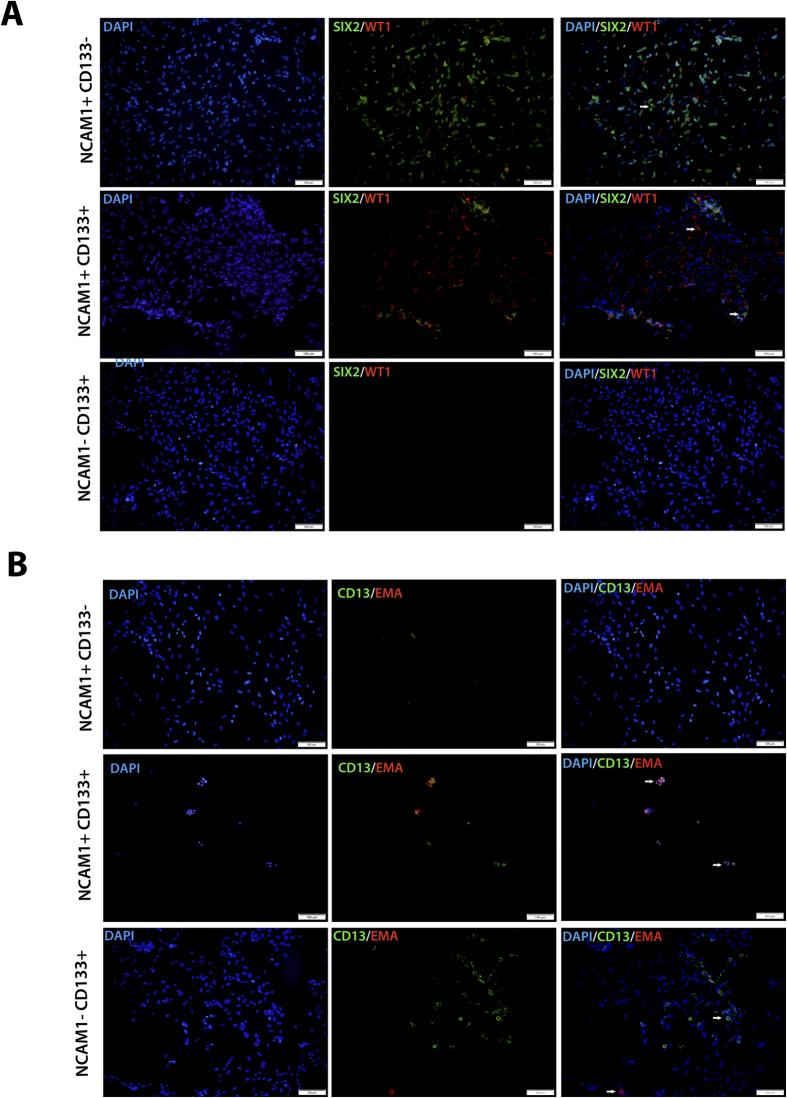
SIX2 is highly expressed by NCAM1^+^CD133− cells, while EMA and CD13 are upregulated in NCAM1^+^CD133^+^ and NCAM1^−^CD133^+^ at the protein level. Sorted cells were fixed on coverslips and immonostained for SIX2, WT1, EMA and CD13. (**A**) Immunofluorescent staining confirms the RNA sequencing and qRT-PCR gene expression results at the protein level showing SIX2 (green) to be highly expressed by NCAM1^+^CD133^−^ cells while few or no cells were stained in the NCAM1^+^CD133^+^ and NCAM1^−^CD133^+^ subpopulations respectively. WT1 (red) was highly expressed by both NCAM1^+^CD133^−^ and NCAM1^+^CD133^+^ cells. (**B**) CD13 (green-marker of proximal tubular cells) showed the highest expression in the NCAM1^−^CD133^+^ and EMA (red- marker of distal tubular cells) was highly expressed by NCAM1^+^CD133^+^ while NCAM1^+^CD133^−^ hFK cells were mostly devoid of their expression.

Having established a typical molecular signature to each of the three hFK-derived fractions at the transcript level, we were interested in further validating these results at the protein level. For this purpose, we carried out immunofluorescent staining (IF) of the three fractions for the CM-related transcription factors, SIX2 and WT1 (Fig. 3A). Consistent with the RNA-sequencing results, we detected strong SIX2 expression in the NCAM1^+^CD133^−^ cell fraction, while NCAM1^+^CD133^+^ and NCAM1^−^CD133^+^ cells demonstrated weak and absent expression, respectively. Similarly, WT1 was expressed in NCAM1^+^CD133^−^ and NCAM1^+^CD133^+^, but not in NCAM1^−^CD133^+^ cells. Notably, WT1 expression has been previously shown to persist in later stages of renal differentiation compared to SIX2, which is exclusively expressed during the CM stage. We next stained the cells for the renal differentiation markers CD13 and EMA, representing mature proximal and distal tubular cells, respectively (Fig. 3B). Consistent with their CM-like phenotype, NCAM1^+^CD133^−^ cells did not express any of the markers. In contrast, CD13 and EMA were expressed in both NCAM1^+^CD133^+^ and NCAM1^−^CD133^+^ cells. Taken together, these results confirm the RNA-sequencing data, revealing upregulation of CM-related factors alongside downregulation of differentiation markers in the hFK NCAM1^+^CD133^−^ cell population.

### NCAM1^+^CD133^−^ cells possess multi-lineage differentiation potential

**Figure 4:**
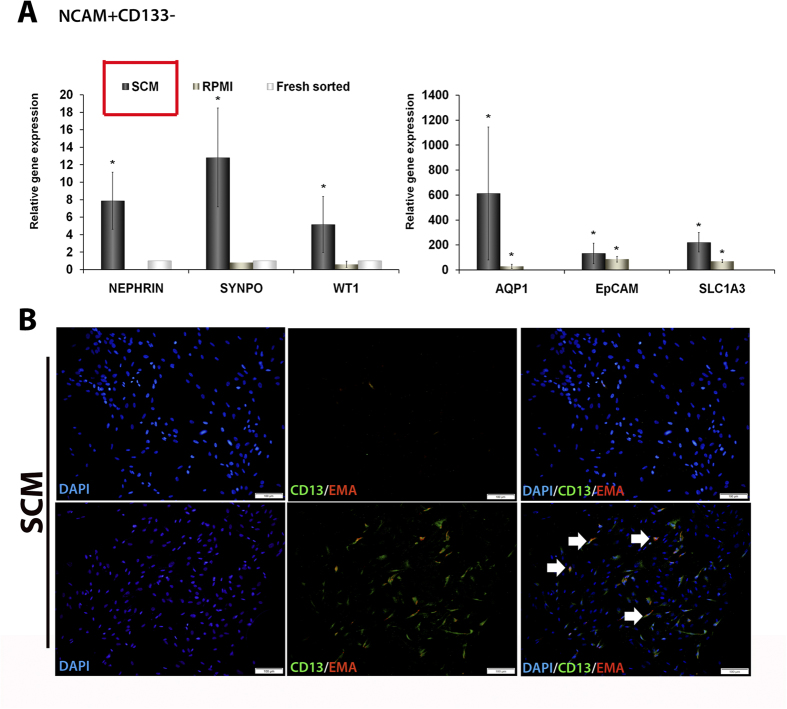
Sorted NCAM1^+^CD133^−^ hFK cells show differentiation potential towards different compartments of the mature human kidney when grown in specific conditions. Sorted NCAM1^+^CD133^−^ hFK cells were cultured in two types of media: RPMI based serum containing medium (RPMI) or IMDM based serum containing medium (SCM), for 10 days. (**A**) qRT-PCR was performed on cultured cells comparing the expression of podocyte (Nephrin, Synaptopondin and WT1), proximal tubular (AQP1) and distal tubular (SLC1A3 and EpCAM) markers between the two culture conditions in comparison to freshly sorted NCAM1^+^CD133^−^ cells. All markers were most highly expressed by cells cultured in SCM compared to sorted fresh cells. The values for NCAM1^+^CD133^−^ fresh sorted cells were used to normalize (therefore = 1) and all other values were calculated with respect to them. Experiments were performed on 2 hFK sources (n = 2). Results are presented as the mean ± S.E.M of three separate experiments; *p < 0.05; (**B**) Immunofluorescence staining for proximal tubular (CD13) and distal tubular (EMA) differentiated epithelia markers was performed on NCAM1^+^CD133^−^ sorted cells grown in each condition for 10 days. Upper panel: freshly sorted NCAM1^+^CD133^−^ cells do not express CD13 and EMA, while lower panel shows both to be highly expressed by NCAM1^+^CD133^−^ cells after culturing in SCM (note that some cells show double staining for both markers suggesting an intermediate differentiation state –white arrows) in consensus with the gene expression results.

In order to further test the hypothesis that the NCAM1^+^CD133^−^ fraction of hFK represents a subpopulation of renal progenitors, we evaluated their ability to differentiate towards several types of epithelial renal lineages. For this purpose, we cultured sorted NCAM1^+^CD133^−^ cells for 10 days in RPMI-based serum-containing medium (RPMI) or IMDM-based serum-containing medium (SCM). First, we compared the expression of podocyte (Nephrin, Synaptopondin and WT1), proximal tubular (AQP1) and distal tubular (SLC1A3 and EpCAM) markers in cells cultured in each of the media types to freshly sorted cells, via qPCR analysis. NCAM1^+^CD133^−^ cells cultured in SCM demonstrated significant upregulation of all markers, compared to freshly sorted cells, while those cultured in SFM showed predominant elevation of the distal tubular epithelial gene EpCAM (Fig. 4A). Next, we asked whether NCAM1^+^CD133^−^ cells cultured in SCM would demonstrate up-regulation of CD13 and EMA, markers of differentiated proximal and distal tubular cells, respectively. Indeed, IF revealed that upon 10 days of culture in SCM, NCAM1^+^CD133^−^ cells, previously shown to be devoid of the expression of CD13 and EMA (Figs 3 and 4B upper panel), exhibited expression of both markers (Fig. 4B). Taken together, both gene expression and immunostaining demonstrate that NCAM1^+^CD133^−^ cells harbor multipotency towards several renal epithelial lineages.

### NCAM1^+^CD133^−^ cells demonstrate a WT blastema phenotype and are capable of giving rise to differentiated WT elements

**Figure 5:**
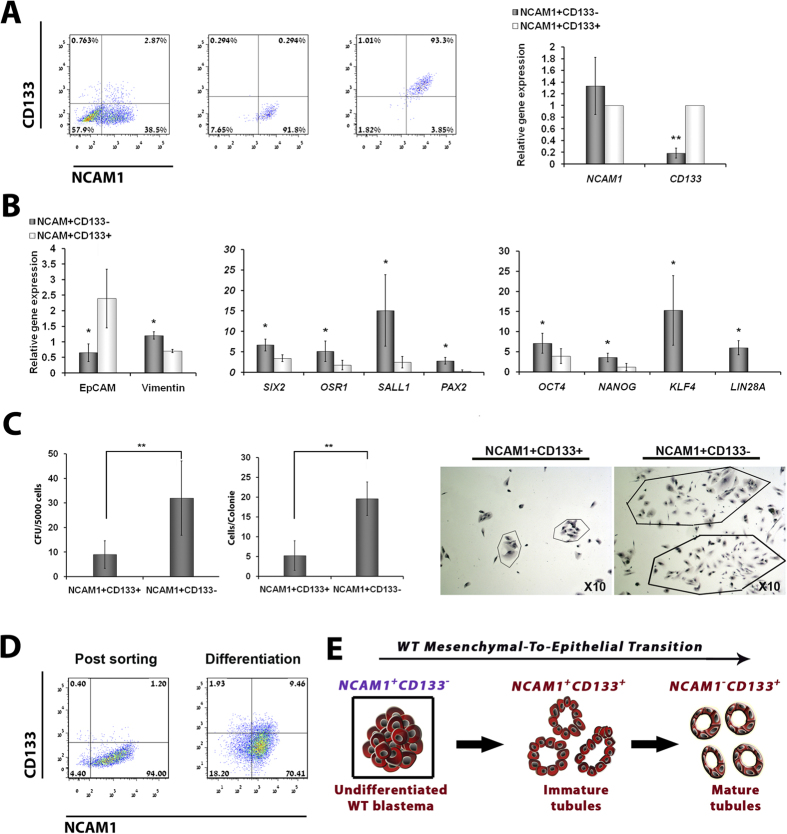
NCAM1^+^CD133^−^ cells demonstrate blastema phenotype in pWT (**A**) Representative FACS sorting of pWT according to NCAM1 and CD133 expression showing the purity of the sorted cell fractions both at the protein (FACS plots of sorted NCAM1^+^CD133^+^ and NCAM1^+^CD133^−^ cells – left) and transcript levels (relative gene expression levels as determined by quantitative RT-PCR [qPCR] analysis). (**B**) qPCR analysis of sorted NCAM1^+^CD133^+^ and NCAM1^+^CD133^−^ showing in the latter a more mesenchymal phenotype (higher *Vimentin* and lower *EpCAM* levels) and higher levels of both renal progenitor- (*OSR1*, *SIX2, SALL1* and *PAX2*) and stemness (*KLF4, LIN28A, OCT4* and *NANOG*) genes. Expression levels in NCAM1^−^CD133^−^ cells were used to normalize (control) and all other values were calculated with respect to them. Experiments were performed on pWT from 5 different donors (n = 5). Results are presented as the mean ± S.E.M of four separate experiments; *p < 0.05; (**C**) Comparison of colony formation capacity between NCAM1^+^CD133^+^ and NCAM1^+^CD133^−^ cells derived from pWT. The number of colonies derived from NCAM1^+^CD133^+^ cells was significantly lower than from NCAM1^+^CD133^−^ cells (left bar graph). Representative phase-contrast images of colonies derived from NCAM1^+^CD133^+^ and NCAM1^+^CD133^−^ cells are presented on the right (magnification = X10). Experiments were performed on 4 pWT tissues (n = 4) and were repeated twice in triplicates; (**D**) NCAM1^+^CD133^−^ sorted primary WT cells were grown in IMDM based serum containing media for 7 days. Next the cells were harvested and analyzed by FACS for the expression of NCAM1 and CD133. Left plot shows sorting purity of the NCAM1^+^CD133^−^ fraction post sorting (94% and 1.6% of CD133^+^ cells), while the right plot shows NCAM1 and CD133 expression after culturing. NCAM1^+^CD133^−^ cells form CD133^+^ cells (mostly NCAM1^+^CD133^+^ cells −9.5%, but also NCAM1^−^CD133^+^ −1.9% -total 11.4% of CD133^+^ cells) following culture in SCM suggesting they possess epithelial differentiation capacity; (**E**) Schematic representation of the epithelial lineage in WT according to the renal developmental MET, defined by NCAM1 and CD133 expression; the NCAM1^+^CD133^−^ phenotype marks the undifferentiated blastema whereas NCAM1^+^CD133^+^ marks immature tubules and NCAM1^−^CD133^+^ identifies mature tubular epithelia.

Having shown that the NCAM1^+^CD133^−^ phenotype in hFK represents a pool of undifferentiated multipotent cells and that undifferentiated WT elements exhibit a NCAM1^+^CD133^−^ phenotype *in situ*, we next wished to assess whether sorted NCAM1^+^CD133^−^ WT cells harbor a cellular phenotype consistent with a blastemal identity. First, we validated the sorting purity at both the protein and transcript levels. Flow cytometry analysis and quantitative real-time PCR (qPCR) were used to compare the NCAM1^+^CD133^−^ and NCAM1^+^CD133^+^ sorted fractions; (Fig. 5A). Since WT blastema is characterized by a mesenchymal phenotype and by over-expression of CM-related and stemness genes^20^, we next compared the expression of MET-related, CM-related and stemness genes between the two fractions, using qPCR (Fig. 5B). NCAM1^+^CD133^−^ cells demonstrated a more mesenchymal phenotype, as manifested by the significantly increased Vimentin and decreased EpCAM levels, compared to NCAM1^+^CD133^+^ cells. In addition, the NCAM1^+^CD133^−^ fraction displayed significantly higher levels of renal progenitor (i.e. *SIX2, OSR1*, *SALL1* and *PAX2)* and stemness (i.e. *OCT4, NANOG, KLF4* and *LIN28A*) genes with respect to the NCAM1^+^CD133^+^ fraction. Since colony formation capacity is a well-established trait of stem/progenitor cells and has been previously ascribed to WT blastemal cells^20^, we subsequently carried out clonogenicity assays. Indeed, NCAM1^+^CD133^−^ cells gave rise to a significantly higher number of colonies compared to NCAM1^+^CD133^+^ cells. Notably, the colonies established by the former were composed of a significantly greater number of cells than those formed by the latter, reflecting an enhanced colony formation capacity of the NCAM1^+^CD133^−^ cells (Fig. 5C). Finally, we wished to evaluate whether the undifferentiated NCAM1^+^CD133^−^ fraction of pWT harbors epithelial differentiation potential. For this purpose, we cultured sorted NCAM1^+^CD133^−^ pWT cells in SCM for 7 days, after which the cells were analyzed for the expression of NCAM1 and CD133 via flow cytometry (Fig. 5D). Interestingly, the NCAM1^+^CD133^−^ fraction gave rise to both NCAM1^+^CD133^+^ cells and NCAM1^−^CD133^+^ cells. Taken together, these results indicate that the NCAM1^+^ CD133^−^ cell fraction in pWT corresponds to the undifferentiated blastemal compartment possessing a mesenchymal phenotype, typical gene expression and *in vitro* colony formation capacity. In addition, this undifferentiated fraction is capable of giving rise to the more differentiated epithelial fractions of WT (Fig. 5E).

### WT-CSCs reside within the NCAM1^+^CD133^−^ blastemal compartment of primary WT and maintain their epithelial committed phenotype in WT PDX

**Figure 6:**
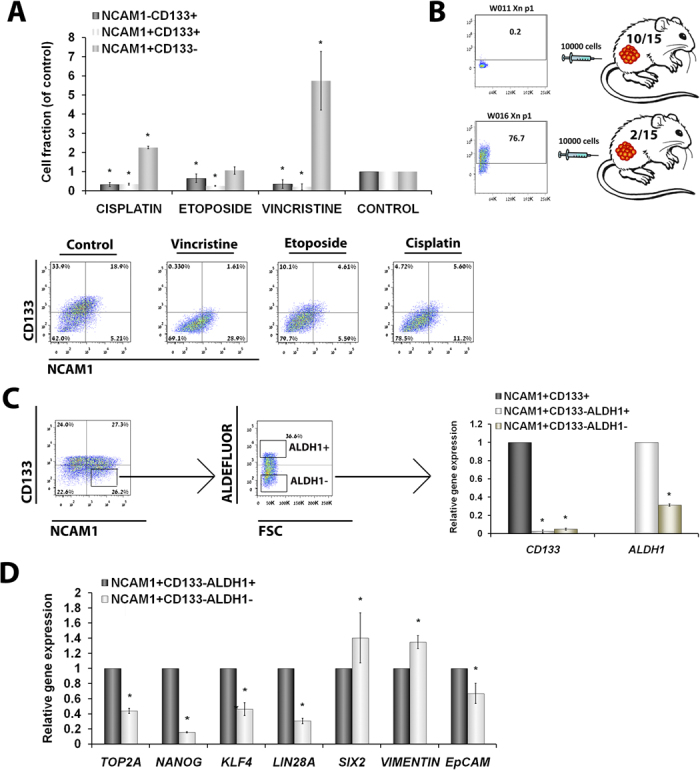
Wilms’ tumor CSCs reside within the NCAM1^+^CD133^−^ blastema. (**A**) Effects of conventional first- (Vincristine) and second-line (Etoposide and Cisplatin) chemotherapies on the percentage of CD133^+^NCAM1^−^, NCAM1^+^CD133^+^ and NCAM1^+^CD133^−^ pWT cells, representing tubular epithelia, immature tubules and blastema, respectively, compared to vehicle-treated control cells, as assessed by flow cytometry. Top: Vincristine and Cisplatin treatment significantly increases NCAM1^+^CD133^−^ fraction, while Etoposide has no effect. In contrast, the NCAM1^−^CD133^+^ and NCAM1^+^CD133^+^ fractions significantly decrease upon treatment. Bar graph summarizing experiments from 3 different pWT sources (n = 3). p < 0.05 relative to control group was considered significant. The percentages of subpopulations in the untreated control group were used to normalize (therefore = 1). Bottom: Representative flow cytometry plots of pWT cells treated with first- or second line chemotherapies, showing an increased percentage of NCAM1^+^CD133^−^ cells following treatment with Vincristine or Cisplatin and a reduced percentage of NCAM1^−^CD133^+^ and NCAM1^+^CD133^+^ cells in all treated cultures, compared to untreated cells; (**B**) CD133 expression in first passage WT-Xn limits *in vivo* propagation. A scheme depicting the experiments, showing representative FACS plots of WT-PDX with low (top) and high (bottom) CD133 expression. Following injection of 10^4^ WT-PDX cells into the flanks of NOD-SCID mice (n = 15 mice per tumor) graft take was 67% for the WT-PDX with low CD133 expression (top) and 13% for WT-PDX expressing high CD133 levels (bottom); (**C**) Representative FACS sorting of pWT according to NCAM1 and CD133 expression and ALDH1 activation showing the purity of the sorted fractions at the protein (FACS plots of sorted NCAM1^+^CD133^−^ALDH1^+^ and NCAM1^+^CD133^−^ALDH1^−^ cells – left) and transcript levels (relative expression as determined by qPCR analysis). (**B**) qPCR analysis of sorted NCAM1^+^CD133^−^ALDH1^+^ and NCAM1^+^CD133^−^ALDH1^−^ cells, showing in the former higher expression of stemness and poor WT prognostic genes (*KLF4, LIN28A,* , *NANOG* and *TOP2A respectively*). Within the NCAM1^+^CD133^−^ fraction, NCAM1^+^CD133^−^ALDH1^+^ cells demonstrate a more epithelial phenotype (higher EpCAM and lower Vimentin and SIX2 expression) than the NCAM1^+^CD133^−^ALDH1^−^ cells. The values for NCAM1^+^CD133^−^ALDH1^+^ cells were used to normalize (therefore = 1). Experiments were performed on 2 pWT sources (n = 2). Results are presented as mean ± S.E.M of three separate experiments; *p < 0.05; Figure drawn by Naomi Pode-Shakked.

Recently, we demonstrated that the blastemal NCAM1^+^ALDH1^+^ cell fraction represents the WT CSCs in a WT-PDX model^20^. We next zoomed in on WT blastema using a unique WT-PDX model. This was achieved by implantation of primary heterogeneous WT into immunodeficient mice and propagation of the tumor cells for many generations resulting in a selection of the undifferentiated blastema and disappearance of the differentiated tumor elements giving rise to pure blastema WT PDXs. Close analysis of these tumors revealed that the NCAM1^+^SIX2^+^ homogenously appearing blastema is in fact heterogeneous. Interestingly, we have found that there exists a small scale MET process within the WT blastema and that the NCAM1^+^ALDH1^+^WT CSCs, are not as one would suspect the least differentiated cells, but rather are arrested at a specific stage along the renal MET axis^21^. Having established this in the pure blastema WT-PDX model we next asked whether the CSCs maintain their phenotype in primary WTs^20^. Thus, we were interested in localizing WT CSCs in respect to the blastemal NCAM1^+^CD133^−^ population. If NCAM1^+^CD133^−^ cells contain the WT CSC they should harbor CSC traits. Therefore, we initially evaluated their relative sensitivity to the first- and second line chemotherapeutic agents clinically used to treat patients with WT (Fig. 6A). Application of vincristine or cisplatin to heterogeneous pWT cells resulted in a significant increase in the percentage of NCAM1^+^CD133^−^ cells, compared to control cells, as determined by flow cytometry analysis. In contrast, vincristine, cisplatin and etoposide, all led to a significant decrease in the percentage of the NCAM1^−^CD133^+^ (mature epithelia) and NCAM1^+^CD133^+^ (immature epithelia) fractions. Hence, NCAM1^+^CD133^−^ WT blastemal cells demonstrate relative resistance to chemotherapeutic agents, a hallmark of CSCs. As previously shown, CD133 expression in pWT and mainly in propagatable WT-PDX is relatively low, usually below 10%^13^. In addition, the efficiency of graft take during WT-PDX propagation is approximately 80%^13^. Interestingly, when we attempted to propagate first generation WT-PDX expressing high CD133 levels (i.e. 77%), graft take was approximately 13% under the same conditions (Fig. 6B), suggesting that CD133^+^ cells are devoid of tumor formation capacity. Recently, we utilized the pure blastemal WT-PDX model to show that within the blastema, NCAM1^+^ALDH1^+^CSCs do not correspond to the earliest renal stem cells but are rather phenotypically committed epithelial progenitors^21^. So as to assess whether the CSCs in pWT possess the same phenotype as in WT-PDX, we sorted pWT cells into NCAM1^+^CD133^−^ALDH1^+^ and NCAM1^+^CD133^−^ALDH1^−^ blastemal fractions (Fig. 6C). Indeed, qPCR analysis of the sorted subpopulations revealed the NCAM1^+^CD133^−^ALDH1^+^ cells to overexpress the characteristic WT-CSC gene-set, consisting of stemness and poor prognostic genes (i.e. *KLF4, LIN28A NANOG* and *TOP2A*, respectively)^13^,^30^ (Fig. 6D). In addition, this fraction demonstrated a more epithelial phenotype compared to NCAM1^+^CD133^−^ALDH1- blastemal cells (i.e. higher *EpCAM* and lower *Vimentin* and *SIX2* levels), as previously shown in pure blastema WT Xn^21^. Taken together, our results imply that like in the WT-Xn models, CSCs in pWT reside within the NCAM1^+^CD133^−^ blastema and harbor a partially committed epithelial progenitor phenotype in the blastema, transiting between more epithelial and early mesenchymal states thereby forming the heterogeneous tumor phenotype.

## Discussion

Utilizing two surface markers, we have marked consecutive stages of nephrogenesis and kidney tumorigenesis, describing a simple method for the prospective isolation of nephron stem cells. Previously, utilizing NCAM1 immunosorting and serum free culture we obtained two subpopulations of earlier stem (NCAM1^+^SIX2^+^) and more differentiated epithelial progenitors (NCAM1^+^SIX2^−^)^23^.

We used serum free media (SFM) based culture to enrich for nephrogenic lineage over stromal lineage that appears to expand under serum containing media (SCM)^23^. In this work we show that in low passage hFK cell cultures grown in SFM, the interstitial cells are eliminated while CM and epithelial lineages are maintained (Figure S3). Utilizing these interstitial-free hFK cell cultures we show for the first time, that negative selection of CD133 of the entire NCAM1 expressing population selects for a very high percentage of NCAM1^+^SIX2^+^ cells. Accordingly, this combination of prospective isolation and specific serum free culture conditions eliminates the need for using yet additional surface markers such as FZD7 for selection of a pure nephron stem cell population. In primary WTs we have previously showed that SCM preserves the heterogeneous cell populations (blastema, epithelia and stroma) and therefore is a preferable media for their culture^13^.

**Figure 7:**
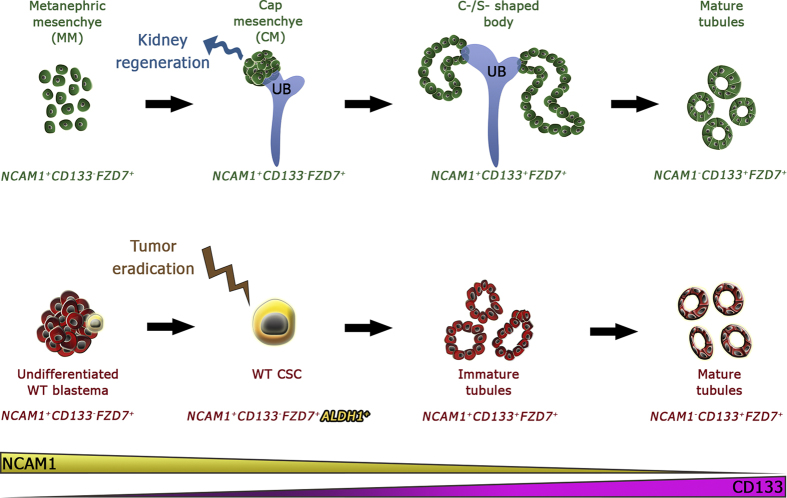
pWT and hFK lineage hierarchy according to SIX2, NCAM1, CD133, FZD7 and ALDH1. The scheme demonstrates the cellular phenotypes along the renal mesenchymal to epithelial transition (MET) axis in both the human developing kidney (upper row) and primary human WT. Along this axis, NCAM1 expression gradually decreases, while that of CD133 increases. FZD7 is expressed throughout the renal MET process, but, not on all cell types in the different compartments. It is highly expressed by some of the hFK condensed mesenchyme (CM) and WT blastema cells. hFK CM progenitors (NCAM1^+^CD133^−^FZD7^+^ or NCAM1^+^CD133^−^ in hFK cultured in SFM) and WT CSCs (NCAM1^+^CD133^−^ALDH1^+^) phenotypes are indicated. Possible isolation or targeting of these two populations, based on the markers described in this work, could facilitate kidney regeneration or WT eradication, respectively. For Figures S1–S5 and Table S1-see Supplemental Data doc.

In both hFK and primary WT, we initially show by means of immunohistochemical staining and flow cytometry analyses, that several distinct cell populations could be identified, distinguishable from one another by the expression of NCAM1 and CD133: The first, NCAM1^+^CD133^−^ population, corresponded to the undifferentiated CM and WT blastema as well as to the renal interstitium (which is eliminated in SFM hFK cell cultures) and is actually interchangeable with the CM SIX2^+^ cells. The second, NCAM1^+^CD133^+^, corresponded to early post-MET structures (e.g. C/S- shaped bodies and immature tubules in hFK and WT, respectively). Third, NCAM1^−^CD133^+^, corresponded to differentiated tubular epithelia. The significance of this sequential mapping of cell sub-populations is in that NCAM1 and CD133 display opposite expression gradients along the renal MET axis in development and tumorigenesis: While NCAM1 expression dominates the undifferentiated, mesenchymal structures and is gradually lost along epithelial differentiation, CD133 expression increases concomitant with renal epithelialization, and overlapping expression of both NCAM1 and CD133 represents the early post-MET structures. This phenotypical hierarchy harbors functional consequences; in the case of nephrogenesis, *in-vitro* differentiation potential analysis of hFK sorted cell subpopulations reveals that NCAM1^+^CD133^−^ cells retain multipotency as shown via gene expression and immunofluorescent staining, demonstrating significant upregulation of diverse renal differentiation markers following their incubation, mostly in SCM (Fig. 4). Moreover, as the differentiation ensues into NCAM1^+^CD133^+^ or NCAM1^−^CD133^+^ lineage restriction is apparent in accordance with our *in vivo* findings for post-MET kidney cell growth^8^. In the case of tumorigenesis, *in vivo* xenotransplantation assays showed that CD133 positivity restricted tumor initiation capacity and hence WT CSCs function. Indeed, the NCAM1^+^ALDH1^+^WT CSC population previously discovered in early and late passage WT-PDX^20^,^21^ is located in the blastema of primary WT within the NCAM1^+^CD133^−^ WT cell fraction and outside of the CD133 expression domain. Thus, the presence and exact localization of the WT CSC is further confirmed in primary WT. An additional layer to our findings is observed in RNA sequencing analysis performed. Results of these studies clearly underscored a strong predominance of expression of CM-related and mesenchymal genes in the NCAM1^+^CD133^−^ cells (e.g. SIX2, OSR1 and EYA1), and an opposite gradient of epithelial gene expression upregulated in NCAM1^+^CD133^+^ and NCAM1^−^CD133^+^ cells (e.g. CDH1 and EpCAM). This gradual pattern, switching from multipotent to differentiated qualities along the hierarchical MET sequence was further supported by splice isoform switching in the genes CD44, ENAH, and CTNND1, similar to what was previously observed in epithelial-mesenchymal transition (EMT) in embryonic development and metastatic breast cancer^24^,^25^,^26^,^27^,^28^,^29^. Finally, single cell qPCR gene expression analysis showed the CD133^+^ hFK fraction (but not the NCAM1^+^ fraction) to expresses epithelial markers such as CDH1 (Fig. 7). Collectively, our findings may bear translational consequences; for nephron regeneration it is tempting to speculate that the highly SIX2-expressing homogenous NCAM1^+^CD133^−^ fraction established in serum-free conditions would be more efficacious than the more heterogeneous NCAM1^+^ cells^1^,^7^,^23^. Second, having previously established the WT NCAM1^+^ALDH1^+^CSC phenotype in pure blastema WT-PDX we have now demonstrated that they share the same phenotype with the primary WT NCAM1^+^CD133^−^ALDH1^+^CSCs, thereby validating our WT-PDX model for studying WT tumorigenesis and maintenance.

Finally, while our pre-clinical findings in WT-PDX showing how CSC targeted therapy with an anti-NCAM1 immunoconjugate eradicated the PDX^20^,^21^ and this form of therapy is currently evaluated in a clinical trial for relapsing WT, finding ways to target an even more specific WT cell population could be promoted by our novel findings. The more precise characterization of the WT CSCs (Fig. 7) could advance elucidation of highly specific novel drug targets allowing the development of more effective targeted treatment strategies with less adverse effects against human WT.

## Experimental Procedures

### Ethics statement

This study was conducted according to the principles expressed in the Declaration of Helsinki and was approved by the Institutional Review Boards of Sheba, Hadassah-Ein Kerem and Asaf Harofeh Medical Centers.

### Human fetal kidney (HFK) and primary Wilms’ tumor (WT) samples

Primary WT samples were obtained from 20 patients within 1hr of surgery from both Sheba Medical Center and Hadassah-EinKerem hospital. HFK were collected from elective abortions. Fetal gestational age ranged from 15 to 19 weeks. All studies were approved by the local ethical committee and informed consent was given by the legal guardians of the patients involved according to the declaration of Helsinki.

Each tissue was processed for immunohistochemical (IHC) staining and formation of single cell suspension as previously described^20^. For patient characteristics see also Supplementary Table S1.

### Primary WT and HFK cell cultures

Were performed as previously described^12^,^20^,^23^. Single cells suspensions of primary WTs and HFK were resuspended in a growth medium (*either serum-containing or serum-free medium*s) and plated in flasks. Serum containing medium (SCM) was comprised of IMDM (Biological Industries) supplemented with 10% foetal bovine serum (Invitrogen), 1% Pen–strep 100 M, 1% l-glutamine (both from Biological industries), 100 ng/ml EGF, 100 ng/ml bFGF and 10 ng/ml SCF (all growth factors purchased from Peprotech Asia). For passaging, cells were detached using 0.05% trypsin/EDTA (Invitrogen). Serum-free medium (SFM) was comprised of N2 medium (Biological Industries) supplemented with 1% Pen–strep 100 M, 1% l-glutamine, 0.4% B27 supplement (Gibco), 4 μg/ml heparin sodium (Intramed), 1% non-essential amino acids, 1% sodium pyruvate, 0.2% CD lipid concentrate (all from Invitrogen), 2.4 mg/ml glucose, 0.4 mg/ml transferrin, 10 mg/ml insulin, 38.66 μg/ml putrescine, 0.04% sodium selentine, 12.6 μg/ml progesterone (all from Sigma–Aldrich), 10 ng/ml FGF and 20 ng/ml EGF. For assessment of the differentiation capacity of HFK NCAM^+^CD133^−^ cells, apart from SCM and SFM, cells were cultured in RPMI media comprised of RPMI-1640 (Biological Industries) supplemented with 10% foetal bovine serum (Invitrogen), 1% Pen–strep 100 M, 1% l-glutamine (both from Biological industries). For passaging, cells were detached using non-enzymatic cell dissociation solution (Sigma–Aldrich). Cells were incubated as described previously^13^. All assays were conducted with low-passage cultured cells (passage 0 and passages 0 to 2 for HFK and primary WT respectively). The cells were observed using Nikon Eclipse TS100 and Nikon Digital Sight cameras.

### Fluorescence-activated cell sorting (FACS) analysis

FACS was performed on either freshly dissociated or cultured cells originating from at least 3 independent samples of HFK (ranging from 15 to 19 weeks of human gestation) and 10 independent pWTs samples as previously described^10^. For freshly dissociated cells, small tumor pieces/HFK tissue were dissociated into single cells, washed in RBCs lysis solution (comprised of: 8.3 g NH4Cl, 1.0 g KHCO3, 1.8 ml of 5% EDTA in double distilled H2O) at 1 ml/5 ml cell suspension ratio was applied for 2 min in 4 °C. Cells were then filtered through a 30 μm nylon mesh before final centrifugation. For cultured cells, cells were harvested using 0.05% trypsin/ethylenediaminetetraacetic acid (Gibco, Grand Island, NY, USA) or non-enzymatic cell dissociation solution (Sigma-Aldrich, St. Louis, MO, USA) and a viable cell number was determined using Trypan blue staining (Invitrogen). Cells (1 × 105 in each reaction) were suspended in 50 μl of FACS buffer [0.5% BSA and 0.02% sodium azid in PBS (Sigma-Aldrich and Invitrogen, respectively)] and blocked with FcR Blocking Reagent (MiltenyiBiotec, Auburn, USA) and human serum (1:1) for 15 min at 4 °C. Surface antigens were labeled by incubation with either fluorochrome conjugated -mouse anti-human CD133/1-PE/allophecoaritin (APC) (Miltenyi Biotech, BergischGladbach, Germany) and mouse anti-human NCAM-APC (Biolegend, San Diego, California, USA) or biotinylated - rat anti-human FZD7 (R&D Systems, Minneapolis, MN, USA) primary antibodies, for 45 min. in the dark at 4 °C to prevent internalization of antibodies. When biotinylated primary antibodies were used, after a washing step, the cells were incubated for 20–30 min. with fluorochrome conjugated strepavidin in addition to 7-amino-actinomycin-D (7AAD; eBioscience) for viable cell gating. All washing steps were performed in FACS buffer. Quantitative measurements were made from the cross point of the IgGisotype graph with the specific antibody graph. See also Supplemental Experimental Procedures.

### Assessment of the percentage of cells with high ALDH1 enzymatic activity

Detection of cells with high ALDH1 enzymatic activity was performed using the ALDEFLUOR kit (StemCell Technologies, Durham, NC, USA) as previously described^31^,^32^. Cells were suspended in Aldefluor assay buffer containing BODIPY-aminoacetaldehyde (BAAA), an uncharged ALDH1 substrate followed by incubation for 30–45 min at 37 °C, in the dark. BAAA is taken up only by living cells through passive diffusion and then converted intracellular by ALDH1 into BODIPY-aminoacetate, a negatively charged reaction product, which is retained inside cells expressing high levels of ALDH1, resulting in these cells becoming brightly fluorescent. The fluorescent of these ALDH1 expressing cells (ALDH1+) can be detected by the green fluorescence channel (520–540 nm) of the FACSAria (BD Biosciences, San Jose, CA). As a negative control, for each sample of cells an aliquot treated in the same conditions was additionally incubated with diethylaminobenzaldehyde (DEAB), a specific ALDH1 inhibitor. Incubation of cells with the BAAA without the addition of DEAB resulted in a shift in BAAA fluorescence defining the ALDH1^+^ population. Since only cells with an intact cellular membrane could retain the Aldefluor reaction product, only viable ALDH1^+^ cells were identified.

### FACS sorting

Cells were harvested as described above, filtered through a 30 μm nylon mesh before final centrifugation, and then re-suspended in either in a FACS buffer or in an ALDEFLUOR buffer (when both surface antigens – NCAM, CD133 and ALDH1 activity were analyzed). FACSAria was used in order to enrich for cells expressing surface markers and ALDH1 high activity. A 100-μm nozzle (BD Biosciences, San Jose, CA), sheath pressure of 20–25 pounds per square inch (PSI), and an acquisition rate of 1,000–3,000 events per second were used as conditions optimized for WT cell sorting. Single viable cells were gated on the basis of 7AAD, and then physically sorted into collection tubes for all subsequent experiments. Data was additionally analyzed and presented using FlowJo software Bulk.

### RNA sequencing library construction

Bulk total RNA was prepared from ~1.5*10^5^ cells using the Direct-zol RNA MiniPrep kit (Zymo Research) according to the manufacturer’s instructions and stored in −80 °C. RNA was quantified on an Agilent BioAnalyzer (Agilent Technologies) and aliquots of 270–500 ng were made into cDNA libraries using the TruSeq mRNA-Seq library kit (Illumina).

### DNA sequencing

Libraries were sequenced 1 × 50 bases on the Illumina HiSeq 2000 platform.

### Sequence alignment and analysis

Sequence data was analyzed using the protocol by Anders *et al.*^3^. Briefly, raw reads were aligned by TopHat2^4^ to the human hg19 genome. The reference genome and annotation files were obtained from the Illumina iGenomes collection (http://support.illumina.com/sequencing/sequencing_software/igenome.html). Aligned reads were counted byHTSeq^5^. Data normalization and differential gene expression was done by DESeq2^6^.

The GEO series record for the sequencing data is: GSE78502.

### Heat-maps

Genes marking specific lineages of the developing kidney were selected according to the GUDMAP database^7^,^8^. Genes marking epithelial and mesenchymal phenotypes were chosen according to the literature. In order to draw the gene expression heatmap, expression values for each individual gene were standardized by subtracting the mean and dividing by 3 times the standard deviation. Then, all values were truncated into the range −1 … 1 and visualized. All analysis was done with Matlab (Mathworks).

### Gene set enrichment analysis

We used GSEA^9^ to check for enrichment of gene sets from the Molecular Signatures Database (MSigDB) and the GUDMAP database^7^,^8^.

### Splice variant switching

We used DEXSeq^10^ to count the number of reads that align to each exon in selected genes. We selected genes that were found to undergo splice variant switching during EMT^2^,^11^,^12^,^13^. In each sample, the counts per exon were normalized by dividing by the total counts per gene. For selected genes, Integrative Genomics Viewer (IGV)^14^ was used to visualize splice variant switching. Sketches were drawn using FancyGene^15^ and the Exon-Intron Graphic Maker (http://wormweb.org).

### Single cell qPCR

Single cells were sorted by FACS into individual wells of 96 well plates. After cells lysis, mRNA levels were measured by microfluidic single cell qPCR using the Biomark system (Fluidigm, CA) according to the manufacturer’s instructions. This resulted in 48 gene expression values (measured in threshold cycles, Ct) for each one of the cells sorted. We analyzed approximately 160 cells from fetal human kidney after culturing for a single passage. qPCR standard curves were created using serial dilutions of “bulk” RNA containing a mixture of HeLa total RNA and RNA from adult and fetal human kidneys. TaqMan gene expression primers and probes were purchased from ThermoFisher Scientific. We clustered over the following genes: NCAM1 (Assay ID Hs00941830_m1), PROM1 (CD133, Assay ID Hs01009250_m1), and CDH1 (E-Cadherin, Assay ID Hs01023894_m1).

For clustering analysis, we standardized the expression levels of each gene individually by subtracting the mean and dividing by 3 times the standard deviation. Then, all values were truncated into the range [−1, +1]. Clustering was performed using complete linkage and correlation distance (Matlab).

### Quantitative Real Time reverse transcription PCR analysis – Gene expression analysis

Quantitative reverse transcription PCR (qRT-PCR) was carried out to determine fold changes in expression of a selection of genes {MET associate (E-Cadherin, Vimentin), renal progenitor (*SIX2, OSR1*, *SALL1, PAX2*) and stemness (*KLF4, LIN28A, OCT4 and nanog*)} between NCAM^+^CD133^+^ and NCAM^+^CD133^−^ as well as between NCAM^+^CD133^+^ALDH1^+^ and NCAM^+^CD133^−^ALDH1^−^ primary WT sorted cell subpopulations. See also Supplemental Experimental Procedures.

### Immunohistochemical staining of HFK, primary WT and WT Xn

Immunostaining was performed as previously described^33^. See also Supplemental Experimental Procedures.

### Colony forming assay (CFU)

Cells were routinely cultured in IMDM medium supplemented with 10% FBS (“growth medium”). For assessment of colony forming ability (CFU), primary WT NCAM^+^CD133^+^ and NCAM^+^CD133^−^ sorted cells were plated in growth medium on matrigel-coated 24 well plates at 1000 cells/well in triplicates. Medium was changed twice a week. After two weeks, both the number of colonies and the number of cells per colony were determined, and means calculated.

### *In vivo* Xn formation

The animal experiments were performed in accordance with the Guidelines for Animal Experiments of Sheba Medical Center. Initial WT xenografting to 5–8 weeks old, female, nonobese diabetic immunodeficient mice was performed as previously described^10^. Pure blastema, Late passages WT patient derived xenografts (WT-PDX) were formed by serial injections of approximately 10^6^ dissociate cells from freshly retrieved WT Xn. Cells were injected in 100 μl 1:1 serum free medium/Matrigel (BD Biosciences, San Jose, CA).

Tumorigenicity of first generation WT Xn either expressing CD133 or w/o CD133 expression was assessed by injecting 10^4^ cells in 100 μl 1:1 serum free medium/Matrigel (BD Biosciences, San Jose, CA) subcutaneously into the flanks of secondary recipients NOD/SCID mice. Engrafted mice were inspected bi-weekly for tumor appearance by visual observation and palpation and the number of tumors formed was recorded (each first generation WT-PDX was injected into 15 mice). See also Supplemental Experimental Procedures.

### Treatment of WT cells with first and second line chemotherapies

In order to determine the lethal dose for 50% of WT cells (LD50) with each of the studied drugs (first line – vincristine and second line – etoposide and cisplatin), primary WT cells were seeded in 96-well plates at 10^4^ cells/well for 48 hrs. After the indicated time the medium was replaced with medium containing a range of concentrations for each of the drugs evaluated: For Vincristine 1 μM–250 μM were tested, for Etoposide −1 μM–250 μM were tested and for Cisplatin −1 μM–100 μM were tested (both are used as second line chemotherapies in WT treatment protocols). Following 48 h exposure, the MTS proliferation assay was performed in accordance with the manufacturer’s instructions and the lethal dose for 50% of cells (LD50) was determined. LD50 were 40 μM for Vincristin, 40 μM for Etoposide and 10 μM for Cisplatin. All further experiments evaluating the effects of these drugs on WT cells were performed at these concentrations.

### *In vitro* effects of chemotherapeutic drugs on primary WT cell subpopulations

In order to examine the effect of chemotherapeutic drugs used in first and second line regimens for treatment of WT on primary WT cell subpopulations (according to NCAM and CD133 expression), WT cells (from each source) were plated in 4 × 75 T flasks for 72 h. Following the indicated time, medium was removed and replaced by medium containing vincristin, etoposide, cisplatin or growth medium w/o drugs (untreated). The untreated flask was used as the baseline for NCAM and CD133 expression in each tumor examined. After treatment, cells were incubated for 48 h, the medium was removed, cells were harvested using 0.05% Trypsin/EDTA, counted and analyzed by FACS for the percentage of NCAM^−^CD133^+^ (WT mature tubules), NCAM^+^CD133^+^ (immature tubules) and NCAM^+^CD133^−^ (WT blastema) cell subpopulations as described above.

### Statistical analysis

Results are expressed as the mean ± S.E.M, unless otherwise indicated. Statistical differences in gene expression between WT cell populations were evaluated using the non-parametric, one sided Sign test. Statistical differences between additional data groups were determined with Student’s t test. For all statistical analysis, the level of significance was set as p < 0.05 unless otherwise indicated.

## Additional Information

**How to cite this article**: Pode-Shakked, N. *et al.* Dissecting Stages of Human Kidney Development and Tumorigenesis with Surface Markers Affords Simple Prospective Purification of Nephron Stem Cells. *Sci. Rep.* **6**, 23562; doi: 10.1038/srep23562 (2016).

## Supplementary Material

Supplementary Information

## Figures and Tables

**Table 1:** Expression distribution of NCAM1, CD133 and FZD7 in hFK, pWT and pure blastema WT-PDX.

	hFK	pWT	Pure blastema WT PDX
NCAM1			
Undifferentiated Blastema	5 (5)	9 (9)	4 (4)
Immature Tubules	5 (5)	9 (9)	−
Mature Tubules	0 (5)	0 (9)	−
Glomeruli/glomeruli bodies	0 (5)	0 (9)	−
Interstitium/Stroma	5 (5)	0 (9)	−
Other	0 (5)	0 (9)	−
CD133			
Undifferentiated Blastema	0 (2)	0 (5)	0 (4)
Immature Tubules	2 (2)	5 (5)	−
Mature Tubules	2 (2)	5 (5)	−
Glomeruli/glomeruli bodies	2 (2)	3* (5)	−
Interstitium/Stroma	0 (2)	0 (5)	−
Other	0 (2)	0 (5)	−
FZD7			
Undifferentiated Blastema	3* (3)	4* (5)	4 (4)
Immature Tubules	3* (3)	4* (4)	−
Mature Tubules	3* (3)	4* (4)	−
Glomeruli/glomeruli bodies	0 (3)	0 (4)	−
Interstitium/Stroma	0 (3)	0 (4)	−
Other	0 (3)	0 (4)	−

Numbers represent the number of tissues stained with the specific marker at the specific compartment out of the number of tissues stained (in bracket). Abbreviations: hFK – human Fetal Kidney; pWT – primary Wilms’ tumor; pure blastema WT-PDX – pure blastema Wilms’ tumor patient derived xenograft.

^*^Staining distinct cells within this compartment.

**Table 2:** Cell subpopulations in pWT and hFK according to NCAM1, CD133 and FZD7 expression.

pWT cell subpopulation	Primary Wilms' Tumors									
	WOO2	WOO3	WOO4	WOO5	WOO6	WOO7	WOO9	WO10	WO16	hFK
NCAM1^−^CD133^+^	2.95	0.85	29.7	1.6	2.2	3.8	10.2	45.25	18.3	3.4
NCAM1^+^CD133^+^	15.8	1.1	15.1	1.4	0	56.2	16	25.55	12.2	10.2
NCAM1^+^CD133^−^	50.05	13	18.75	9.9	19.7	23.6	23.6	14.75	14.25	36.9
NCAM1^−^CD133^−^	31.2	85	36.45	87.1	78.1	16.4	50.2	14.46	55.25	49.5
CD133^+^FZD7^−^	18.51	26.73	22.2	22.45	0.5	45	N/A	N/A	23.6	30.6
CD133^+^FZD7^+^	2	2.49	0.9	3.1	0.7	5.1	N/A	N/A	4.4	35.7
CD133^−^FZD7^+^	32.6	2.73	2.4	3.15	38	3.5	N/A	N/A	4.4	11.6
CD133^−^FZD7^−^	46.65	68.05	74.5	71.3	60.8	46.4	N/A	N/A	67.5	22.1
NCAM1^−^FZD7^+^	56.9	N/A	3.4	N/A	N/A	2.9	N/A	53.5	N/A	21.9
NCAM1^+^FZD7^+^	21.8	N/A	24.1	N/A	N/A	17.1	N/A	16.9	N/A	7.1
NCAM1^+^FZD7^−^	3.8	N/A	57	N/A	N/A	55.5	N/A	3.3	N/A	27.9
NCAM1^−^FZD7^−^	27.5	N/A	15.5	N/A	N/A	24.5	N/A	26.3	N/A	42.1

*Table portrays average values (%) from at least two independent experiments per tissue source.

**Table 3:** Differentially expressed genes between NCAM1^+^CD133^−^, NCAM1^+^CD133^+^ and NCAM1^−^CD133^+^ human fetal kidney cells presented according to their function.

Function	Gene Symbol	NCAM1^+^CD133^−^	NCAM1^+^CD133^+^	NCAM1^−^CD133^+^
Cap mesenchyme	NCAM1	690.0590849	354.328209	18.08411771
	SIX1	87.21125876	16.87277186	10.33378155
	SIX2	43.60562938	10.54548241	3.444593849
	DACT1	1931.729382	179.273201	102.476667
	DNM3OS	301.9689835	1.054548241	0.861148462
	HOXC11	77.39999215	42.18192964	24.9733054
	HOXD11	570.1436041	498.801318	396.1282926
	EYA1	35.97464424	25.30915779	28.41789925
	MFAP4	433.8760123	0	6.028039235
	OSR1	4.360562938	1.054548241	0.861148462
	TGFBI	20280.97822	13179.74392	9635.390144
	CDH11	4352.931953	58.00015326	1.722296924
	TPM2	6560.46694	2412.806376	2148.565413
	BMP1	4170.87845	2757.64365	2035.754965
	CDH2	6144.03318	3371.390727	1147.9109
	LEF1	676.9773961	36.90918844	15.50067232
	FOXC2	369.557709	259.4188673	118.8384878
	VIM	78199.06531	74338.26916	50735.4228
MET–Mesenchyme	TCF4	4023.709451	583.1651773	286.7624379
	COL1A2	108569.296	372.2555291	134.3391601
	COL3A1	93693.23557	235.1642578	57.69694697
	COL5A2	16921.16448	4700.121511	1081.602469
	FN1	628191.418	158405.8004	146126.5603
	MMP2	2706.819444	697.0563874	99.89322161
	MMP9	3262.791218	18.98186834	18.94526617
	SERPINE1	25803.63119	2593.134125	2699.700429
	SNAI1	391.3605237	124.4366925	130.8945663
	SNAI2	1690.808279	280.5098321	87.83714314
	TIMP1	23503.43424	3226.917618	2678.171717
	TWIST1	207.1267396	10.54548241	7.75033616
	WNT5A	1230.768889	446.073906	241.9827179
	WNT5B	220.2084284	196.1459728	93.00403392
	SPARC	47648.96136	10534.93693	9285.763868
	TGFB1	2932.478576	1632.440677	1262.443646
	TGFB2	2410.301164	1904.514123	1794.633395
	TGFB3	624.6506409	55.89105678	18.94526617
	ZEB1	1743.135034	584.2197256	187.7303648
	ZEB2	1604.687161	80.14566632	13.7783754
	CDH1	3993.18551	9605.879928	17875.72
	CDH3	8149.892131	15930.00573	22027.32
	CLDN4	15945.48852	23601.84418	30463.99
	CLDN7	2412.481445	4797.139949	8785.44
	CRB3	456.7689677	683.3472602	866.32
	DSP	16809.97013	18159.32071	20067.34
MET–Epithelia	EPCAM	6309.734571	10998.93815	15547.17
	GJB3	806.7041435	1661.968028	2445.66
	KRT18	25851.59738	38248.4647	65194.97
	KRT19	20140.35007	27550.0728	38880.85
	KRT8	39472.90586	63610.3499	103051.91
	MIR200A	1.090140734	8.436385929	7.75
	MUC1	751.1069661	1399.385516	2188.18
	OCLN	1126.115379	2008.914399	2672.14
	PKP2	855.7604766	1503.785792	1818.75
	FZD7	1655.923776	760.3292818	697.53
	POU5F1	123.185903	99.12753466	94.73
hESCs	SALL2	1001.839335	979.675316	521.86
	SOX2	9.8112666	7.381837688	6.88918769
