# Supplementary material for: Dissecting Stages of Human Kidney Development and Tumorigenesis with Surface Markers Affords Simple Prospective Purification of Nephron Stem Cells
Source: Sci Rep. 2016 Mar 29;6:23562. doi: 10.1038/srep23562 (PMC4810363; doi:10.1038/srep23562)
Supplement: Supplementary Information [file srep23562-s1.doc]

DISSECTING STAGES OF HUMAN KIDNEY DEVELOPMENT AND TUMORIGENESIS WITH SURFACE MARKERS AFFORDS SIMPLE PROSPECTIVE PURIFICATION OF NEPHRON STEM CELLS

Pode-Shakked Naomi^1,2,3,9,*#^, Pleniceanu Oren^1,2,9,*^, Gershon Rotem^1,2,9^, Shukrun Rachel^1,2,9^, Kanter Itamar^4^, Bucris Efrat^4^, Pode-Shakked Ben^3,5,9^, Tam Gal^4^, Tam Hadar^4^, Caspi Revital^1,2,9^, Pri-Chen Sara^1,6^, Vax Einav^1,2,9^, Katz Guy^1,2,3,7^, Omer Dorit^1,2,9^, Harari-Steinberg Orit^1,2^, Kalisky Tomer^4^, Dekel Benjamin^1,2,8,9#^

^*^ These authors contributed equally to this work

^1^Pediatric Stem Cell Research Institute, Edmond and Lily Safra Children's Hospital, Sheba Medical Center, Tel-Hashomer, Israel

^2^Sheba Centers for Regenerative Medicine and Cancer Research, Sheba Medical Center, Tel-Hashomer, Israel

^3^The Dr. Pinchas Borenstein Talpiot Medical Leadership Program, Sheba Medical Center, Tel-Hashomer, Israel

^4^Faculty of Engineering and Bar-Ilan Institute of Nanotechnology and Advanced Materials (BINA), Bar-Ilan University, Ramat Gan , Israel.

^5^The Danek Gertner Institute of Human Genetics, Sheba Medical Center, Tel-Hashomer, Israel.

^6^The Maurice and Gabriela Goldschleger Eye Research Institute, Sheba Medical Center, Tel-Hashomer, Israel

^7^The Joseph Buchman Gynecology and Maternity Center, Sheba Medical Center, Tel-

Hashomer, Israel

^8^Division of Pediatric Nephrology, Edmond and Lily Safra Children's Hospital,

Sheba Medical Center, Tel-Hashomer, Israel

^9^Sackler Faculty of Medicine, Tel-Aviv University, Tel-Aviv, Israel

**Supplemental data table of content:**

[Supplemental figures and tables 4](#__RefHeading___Toc438731273)

[Figure S1| late generation WT-PDX are solely composed of blastema cells. 4](#__RefHeading___Toc438731274)

[Figure S2| Primary Wilms' Tumor (pWT) and human fetal kidney (hFK) cell subpopulations
according to NCAM, CD133 and FZD7 expression combinations 5](#__RefHeading___Toc438731276)

[Figure S3| Human fetal kidney (hFK) cultured in Serum Free Msedium (SFM) lose the interstitial cells showing either MNF^+^ or SIX2^+^ cells. 6](#__RefHeading___Toc438731278)

[Figure S4| Single cell qPCR gene expression analysis of hFK cells shows NCAM1 and CD133 to mark different cell fractions. 7](#__RefHeading___Toc438731280)

[Figure S5| RNA sequencing reveals splice isoform switching in accordance with hFK mesenchyal to epithelial (MET) process as manifested by NCAM and CD133 expression. 8](#__RefHeading___Toc438731282)

[Table S1| Patient and tumor characteristics 9](#__RefHeading___Toc438731284)

[Supplemental experimental procedures 10](#__RefHeading___Toc438731285)

[In vivo WT xenograft formation 10](#__RefHeading___Toc438731286)

[Antibodies for fluorescence-activated cell sorting (FACS) analysis and sorting 11](#__RefHeading___Toc438731287)

[Quantitative Real Time reverse transcription PCR analysis – Gene expression analysis 11](#__RefHeading___Toc438731288)

[Immunohistochemical staining of HFK, primary WT and WT Xn. 11](#__RefHeading___Toc438731289)

# Supplemental figures and tables

## Figure S1| late generation WT-PDX are solely composed of blastema cells.


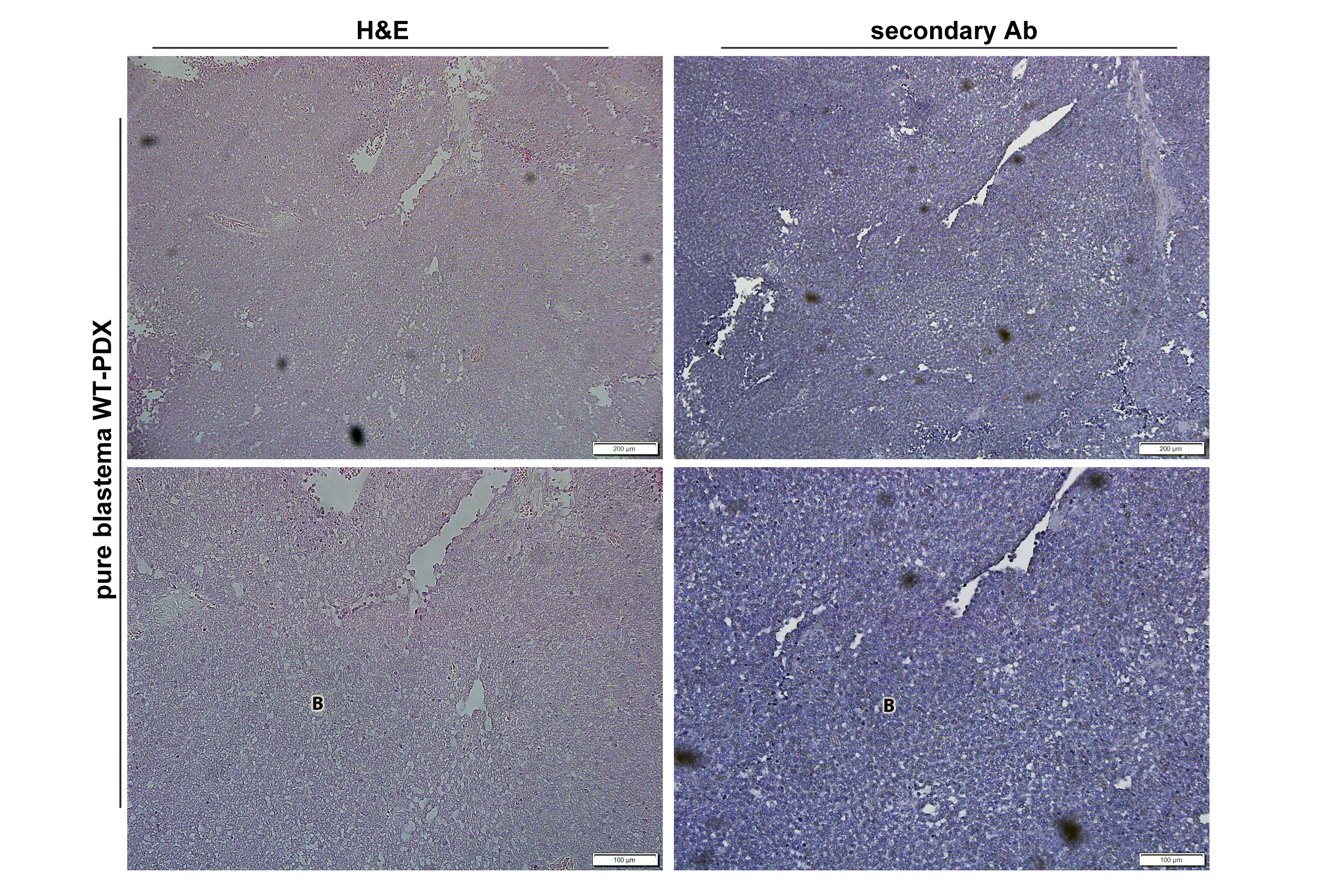


## Representative H&E (left) and secondary antibody only (right) staining of late generation WT-PDX (formed after over 10 passages in immunodeficient mice). These tumors are pure blastemal, the epithelial and stromal elements that where present in their parental tumor disappear. B-Blastema; Scale bars are indicated in the images.

## Figure S2| Primary Wilms' Tumor (pWT) and human fetal kidney (hFK) cell subpopulations according to NCAM, CD133 and FZD7 expression combinations


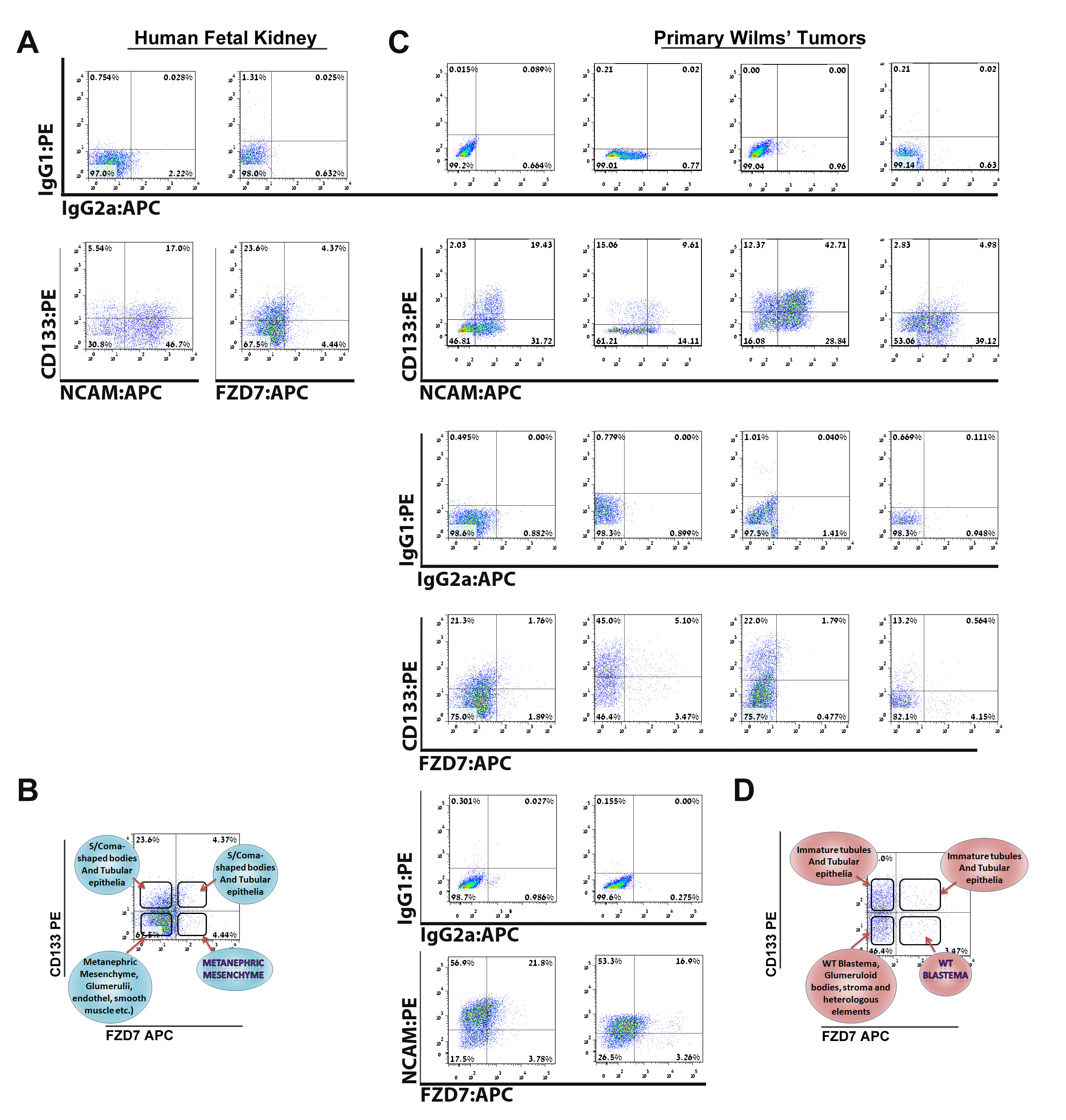


## Representative FACS analyses of NCAM, CD133 and FZD7 expression in hFK (A and B) and primary Wilms' Tumor (pWT) (C and D). Four cell subpopulations can be drawn from expression combinations of these three markers in hFK and pWT respectively – CM/Undifferentiated Blastema (NCAM1+CD133-FZD7+ and partially NCAM+CD133-FZD7-/NCAM1+CD133-); S/Comma shaped bodies/ Immature tubules (NCAM1+CD133+FZD7+ and NCAM+CD133+FZD7-/NCAM1+CD133+); Mature tubules (NCAM1-CD133+FZD7+ and NCAM1-CD133+FZD7-/NCAM1-CD133+), hFK interstitium (NCAM1+CD133-FZD7-) and other non-MET associated structures (NCAM1-CD133-FZD7-). FZD7, although distributed throughout the MET, is not expressed by all cells in each compartment and therefore is inefficient in selecting for all the cells in a specific compartment. Each FACS plot represents a different pWT or HFK.

## Figure S3| Human fetal kidney (hFK) cultured in Serum Free Msedium (SFM) lose the interstitial cells showing either MNF^+^ or SIX2^+^ cells.

##
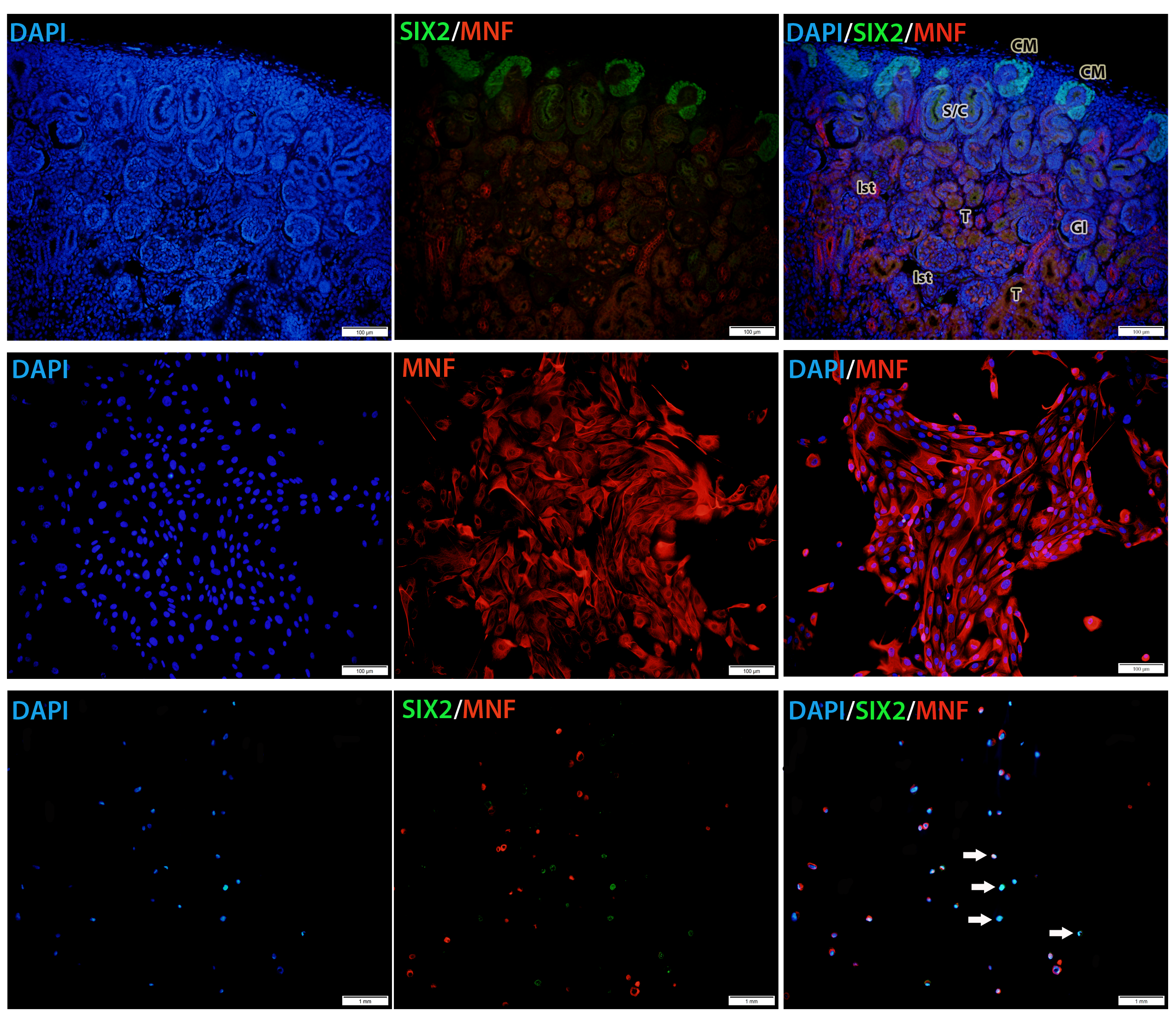


## IF staining for SIX2 and MNF in hFK tissue and cells cultures in SFM. Upper panel - hFK tissue – showing SIX2 to be restricted to the Cap Mesenchyme (CM), while MNF stains mainly tubular epithelial cells (T). The interstitium is not stained with these two markers. Middle panel – hFK cultured in SFM – showing a colony of epithelial cells stained with MNF. Lower panel - hFK cultured in SFM sowing all cells in the culture to be stained either with MNF (epithelia-majority of cells) or with SIX2 (CM-single cells scattered within the epithelial cells) suggesting these cultures are devoid of interstitial cells.

## Figure S4| Single cell qPCR gene expression analysis of hFK cells shows NCAM1 and CD133 to mark different cell fractions.


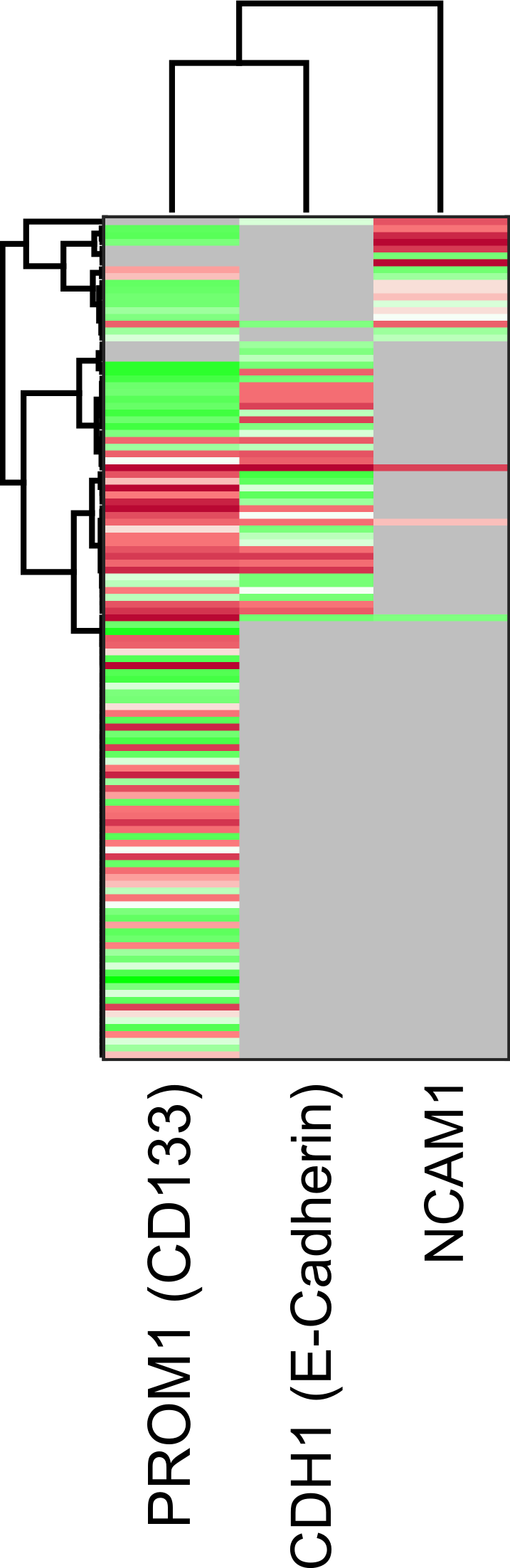


## The CD133^+^ fraction (but not the NCAM1+ fraction) also expresses epithelial markers such as CDH1 (E-Cadherin). Shown are single cell qPCR gene expression measurements from ~160 individual cells. Each column represents a gene while each row represents a single cell. qPCR threshold cycles (Ct’s) were standardized (by subtracting the mean and dividing by 3 times the standard deviation) and clustered (Red – high expression, Green – low expression, Gray – no expression).

## Figure S5| RNA sequencing reveals splice isoform switching in accordance with hFK mesenchyal to epithelial (MET) process as manifested by NCAM and CD133 expression.

##
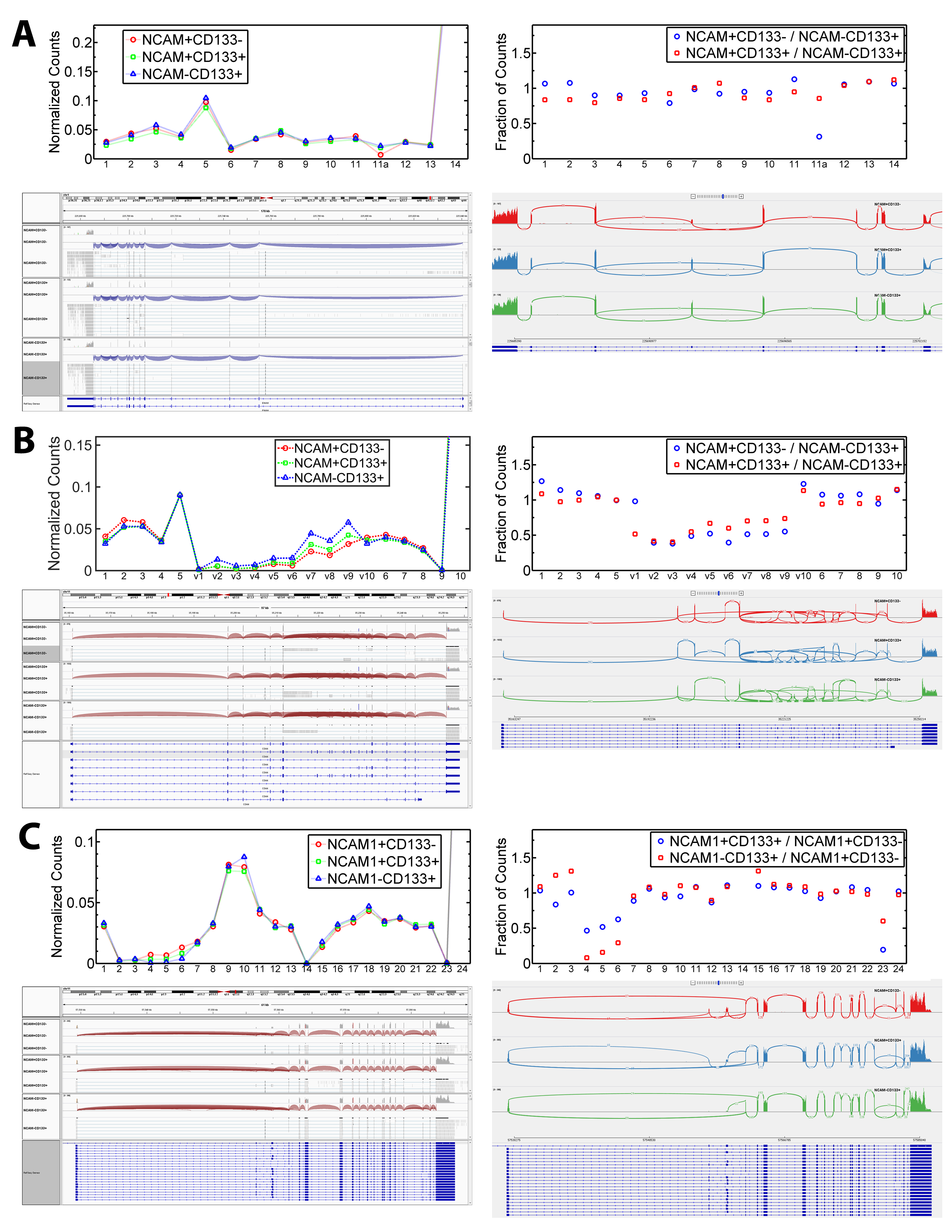


## Splice isoform switching occurs for ENAH, CD44 and CTNND1 in hFK cells crossing from NCAM+CD133- mesenchyme to NCAM+CD133+ and NCAM-CD133+ epithelia. For each gene, shown are the normalized counts (number of RNA sequencing reads) along each exon for each cell fraction (left upper plots), fraction of counts when compared to NCAM+CD133- or NCAM-CD133+ (right upper plots), sashimi plots (right lower plots) and IGV plots (left bottom plots). (A) ENAH has lower counts of the epithelial-associated exon 11a in the NCAM1+CD133- population compared to NCAM+CD133+ and NCAM+CD133- cells.; (B) CD44 has lower counts of the epithelial-associated exons V2-V9 in the NCAM1+CD133- population compared to NCAM+CD133+ and NCAM+CD133- cells; (C) CTNND1 (p120 Catenin) has higher counts of the mesenchymal-associated exons 4,5-6 in the NCAM1+CD133- population compared to NCAM+CD133+ and NCAM+CD133- cells.

## Table S1| Patient and tumor characteristics

| **Remarks** | **Histology** | | **Pattern** | **Age** | **Gender** | **Patient Code** |
| --- | --- | --- | --- | --- | --- | --- |
| Lung metastasis | Favorable histology | | Triphasic | 4 years | Female | W002 |
| Recurrent with diffused anaplasia | | Unfavorable histology | Triphasic -  Blastemal predominance | 10 years | Male | W003 |
| Bilateral | Favorable histology | | Triphasic | 6 years | Female | W004 |
| - | Favorable histology | | Triphasic | 3 years | Male | W005 |
| Focal anaplasia | Favorable histology | | Triphasic | 2 years | Male | W006 |
| Recurrent WT with focal anaplasia | Favorable histology | | Triphasic | 3 years | Female | W007 |
| Recurrent with diffused anaplasia | Unfavorable histology | | Triphasic | 3 years | Male | W009 |
| - | Favorable histology | | Triphasic | 1 years | Female | W010 |
| Recurrent WT with Liver metastasis | Favorable histology | | Triphasic -  Blastemal predominance | 7 years | Female | W011 |
| - | Favorable histology | | Triphasic | 4 years | Male | W013 |
| - | Favorable histology | | Triphasic | 9 months | Male | W014 |
| - | Favorable histology | | Triphasic | 2 rears | Female | W016 |
| - | Favorable histology | | Triphasic | 3 years | Male | W026 |
| - | Favorable histology | | Triphasic | 1 years | Male | W027 |

# Supplemental experimental procedures

## In vivo WT xenograft formation

All animal experiments were conducted in accordance with the National Institutes of Health guidelines for the care and use of animals and with an approved animal protocol from the Sheba medical center Animal Care and Use Committee. Initial WT xenografting to 5-8 weeks old, female, nonobese diabetic immuno deficient mice was performed as previously described (15). Briefly, primary WT tissue was cut into 2-5mm pieces and implanted subcutaneously in the back of the mouse. Tumors were harvested approximately 3-6 month post implantation or when they reached a size of 1.5cm diameter. Single cells suspensions were obtained by mincing the samples in Iscove’s modification of Dulbecco’s medium (IMDM) containing antibiotics (penicillin and streptomycin), followed by treatment with collagenase IV for 2h at 37◦C. Enzymatically treated tissue was triturated using IMDM at twice the volume of the collagenase solution and the suspension filtered (100μm cell strainer) and washed twice with IMDM containing antibiotics. Erythrocytes were removed by ACK RBS lysis buffer.

Tumorigenicity of first generation WT Xn either expressing CD133 or w/o CD133 expression was assessed by injecting 10^4^ cells in 100μl 1:1 serum free medium/Matrigel (BD Biosciences, San Jose, CA) subcutaneously into the flanks of secondary recipients NOD/SCID mice. Engrafted mice were inspected bi-weekly for tumor appearance by visual observation and palpation and the number of tumors formed was recorded (each first generation WT Xn was injected into 15 mice).

Late passages, pure blastema Xn were formed by serial injection of approximately 10^6^ dissociated cells from freshly retrieved WT Xn (p=8-12). Cells were injected in 100μl 1:1 serum free medium/Matrigel (BD Biosciences, San Jose, CA). Engrafted mice were inspected bi-weekly for tumor appearance by visual observation and palpation. Mice were sacrificed by CO_2_ inhalation at a tumor diameter of 1.5 cm or at 6 month post transplantation. Following tumor resection, WT xenograft tumors were fixed and embedded for histological staining of NCAM, SIX2, CD133 and FZD7.

## Antibodies for fluorescence-activated cell sorting (FACS) analysis and sorting

Primary fluorochrome conjugated antibodies: mouse anti-human CD133/1-PE/allophecoaritin (APC) (Miltenyi Biotech, Bergisch Gladbach, Germany); mouse anti-human NCAM-PE (Biolegend, San Diego, California, USA); rat anti-human FZD7-APC (R&D Systems, Minneapolis, MN, USA); In order to visualize the primary unconjugated antibodies, appropriate secondary antibodies were used conjugated to either Alexafluor-488 or Alexafluor-647 (Molecular Probes, Inc., Invitrogen, Eugene, OR, USA).

## Quantitative Real Time reverse transcription PCR analysis – Gene expression analysis

Quantitative reverse transcription PCR (qRT-PCR) was carried out to determine fold changes in expression of a selection of genes {MET associate (E-Cadherin, Vimentin), renal progenitor (*SIX2, OSR1*, *SALL1, PAX2*) and stemness (*KLF4, LIN28A, OCT4 and nanog*)} between NCAM^+^CD133^+^ and NCAM^+^CD133^-^ as well as between NCAM^+^CD133^+^ALDH1^+^ and NCAM^+^CD133^-^ALDH1^-^ primary WT sorted cell subpopulations. Total RNA from cells was isolated using an RNeasy Micro Kit (Qiagen GmbH, Hilden, Germany) according to the manufacturer's instructions. cDNA was synthesized using a High Capacity cDNA Reverse Transcription kit (Applied Biosystems, California USA) on total RNA. Real-time PCR was performed using an ABI7900HT sequence detection system (Perkin-Elmer/Applied Biosystems, California, USA) in the presence of TaqMan Gene Expression Master Mix (Applied Biosystems, California, USA). PCR amplification was performed using gene specific TaqMan Gene Expression Assay-Pre-Made kits (Applied Biosystems, California, USA). Each analysis reaction was performed in triplicate. HPRT1 or GAPDH were used as an endogenous control throughout the experimental analyses. PCR results were analyzed using SDS RQ Manager 1.2 software. Statistical analysis was performed using a non-paired 2-tails T-test. Statistical significance was considered at P<0.05.

## Immunohistochemical staining of HFK, primary WT and WT Xn.

Immunostaining was performed as previously described [4]. Briefly, Sections, 4-µm thick, were cut from HFK (two different kidneys), primary WT (three different tumors) and late, pure-blastema WT Xn (four different sources) for immunohistochemistry. Sections were processed within 1 week to avoid oxidation of antigens. Cuts were mounted on super frost/plus glass (Menzel, Glazer, Braunschweig, Germany) and processed by the labeled – (strept) avidin-biotin (LAB-SA) method using a histostain plus kit (Zymed San Francisco, CA, USA). Heat-induced antigen retrieval was performed by controlled microwave treatment using an H2800 model processor (Energy Bean Sciences, INC) in 10 mM citrate buffer, PH 6.0 for 10 min at 97ºC followed by 3% H2O2 for 10 min. The slides were subsequently stained using the labeled strepavidin-biotin (LAB-SA) method using a Histostain plus kit (Zymed, San Francisco, CA, USA). Anti human SIX2 (ABNOVA, Walnut, USA), anti human NCAM (LifeSpan Biosciences, Inc. Seattle, WA, USA), anti human CD133 () and anti human FZD7 (NOVUS biologicals, Littleton, USA) antibodies were used. Negative control incubations were performed by substituting non-immune serum for the primary antibody. Biotinylated second antibody was applied for 10 min followed by incubation with horseradish peroxidase –conjugated streptavidin (HRP-SA) for 10 min. The immunoreaction was visualized by an HRP-based chromogen/substrate system, including DAB (brown) chromogen (liquid DAB substrate kit – Zymed). The sections were then counterstained with Mayer's hematoxylin, dehydrated and mounted for microscopic examination.
